# Mechanistic Insights into the Anticancer Action of Novel 2‑Hydroxy-1,4-naphthoquinone Thiol Derivatives

**DOI:** 10.1021/acsomega.5c06730

**Published:** 2025-09-05

**Authors:** Thaís Barreto Santos, Alex de Souza Cruz Lopes Canuto, João Francisco Blaudt Virgilio de Carvalho Meira, Ana Caroline Santos-Diniz, Rafaella Machado de Assis Cabral Ribeiro, Caroline Reis Santiago Paschoal, Vitor Won-Held Rabelo, Paula Alvarez Abreu, Vitor Francisco Ferreira, David Rodrigues da Rocha, Bruno Kaufmann Robbs

**Affiliations:** † Institute of Chemistry, 28110 Universidade Federal Fluminense, Niterói, Rio de Janeiro 24020-141, Brazil; ‡ Postgraduate Program in Sciences Applied to Health Products, Faculty of Pharmacy, Universidade Federal Fluminense, Niterói, Rio de Janeiro 24241-000, Brazil; § Postgraduate Program in Morphological Sciences, Institute of Biomedical Sciences, 28125 Universidade Federal do Rio de Janeiro, Rio de Janeiro 21941-590, Brazil; ∥ Department of Basic Sciences, Nova Friburgo Institute of Health (ISNF), Universidade Federal Fluminense, Nova Friburgo, Rio de Janeiro 28625-650, Brazil; ⊥ Postgraduate Program in Pharmaceutical Sciences, Institute of Biodiversity and Sustainability, Universidade Federal do Rio de Janeiro, Macaé, Rio de Janeiro 27965-045, Brazil; # Institute of Biodiversity and Sustainability, Universidade Federal do Rio de Janeiro, Macaé, Rio de Janeiro 27965-045, Brazil; ¶ Department of Pharmaceutical Technology, Faculty of Pharmacy, Universidade Federal Fluminense, Niterói, Rio de Janeiro 24241-000, Brazil

## Abstract

Oral cavity cancer, with squamous cell carcinoma (SCC) representing more than 90% of cases, remains a significant global public health challenge. Novel molecules are urgently needed to combat SCC while reducing adverse effects compared with current therapies. Naphthoquinones, a subgroup of quinones, exhibit diverse pharmacological activities, including antibacterial, antifungal, antiviral, anti-inflammatory, antiparasitic, and anticancer effects. In this study, we evaluated the cytotoxic potential of ten derivatives of α-xyloidone combined with thiols against oral SCC cell lines (SCC-4, SCC-9, and SCC-25). Although eight of these new thionaphthoquinone derivatives were effective against the sensitive SCC-9, only two compounds (**7a** and **7e**) achieved a selectivity index (SI) > 2 against all SCC cell lines. Further evaluation in colorectal (HCT-116), liver (HepG2), and melanoma (B16-F10) cancer models confirmed high selectivity (SI > 2). Both compounds caused less than 2% membrane rupture at concentrations nearly 20-fold above their IC_50_ values, and acute toxicity tests in mice showed no morbidity or mortality. Morphological analysis and caspase activity indicated cell death induced by apoptosis and accompanied by autophagy while inhibiting cell migration efficiently. *In silico* studies predicted favorable human oral bioavailability for **7a**, and both derivatives are expected to inhibit Pg-P with a low predicted toxicity (LD_50_). Docking analyses suggest that **7e** targets key tumor progression enzymes including RSK2 and topoisomerases IIα/IIβ. These findings underscore the cytotoxic potential and safety of thionaphthoquinones **7a** and **7e**, highlighting their promise for oral cancer drug development.

## Graphical abstract

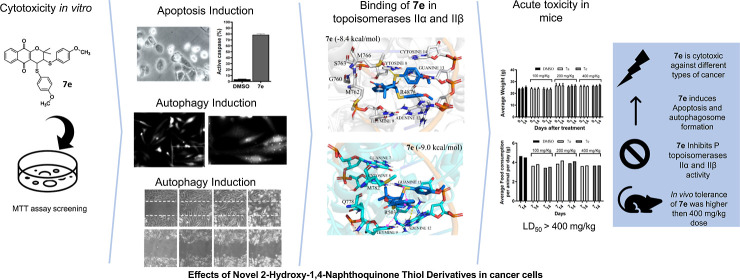

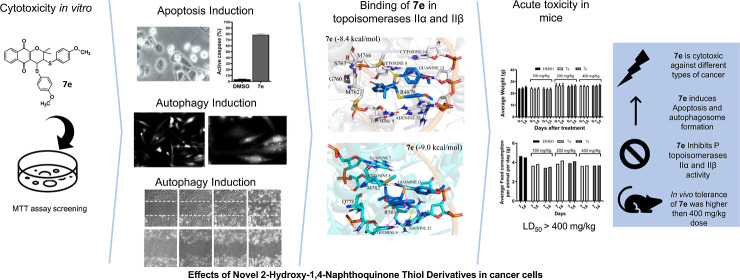

## 1 Introduction

Oral squamous cell carcinoma (OSCC) is the most common histological type of malignant neoplasm of the oral cavity, originating in the epithelium lining the mouth, affecting the lips and the oral cavity itself, and accounting for 90 to 95% of cases of malignant lesions in this region.^1,2^ According to the latest report released by the Global Cancer Observatory, oral cancer (lips and oral cavity) ranks 16^th^ among the thirty-three types of cancer with the highest incidence in both sexes and all ages in the world, with 389,846 new cases, and 15^th^ place in lethality, with 188,438 deaths.^3^

Traditional treatment of OSCC includes several approaches such as surgery, radiotherapy, and chemotherapy, depending on the stage of the disease.^4,5^ The most cited chemotherapy agents for the treatment of OSCC in the clinic in the literature are platinum-based products such as carboplatin, paclitaxel, docetaxel, 5-fluorouracil (5-FU), hydroxyurea, etoposide, pembrolizumab, nivolumab, and cetuximab.^6^ However, the adverse reactions that develop during treatment are well-known: nephrotoxicity, cardiotoxicity, polyneuropathy, and alopecia, to name a few.

Naphthoquinones are secondary metabolite molecules of various living beings such as plants, fungi, bacteria, and some animals, which perform vital biological functions. Several experimental studies have demonstrated its anti-inflammatory, antifungal, antiviral, and antiparasitic actions. Furthermore, other activities have been described, such as anticarcinogenic and even hypoglycemic.^7–11^ Its mechanism of action mainly involves damage to DNA through the production of reactive oxygen species (ROS), inhibition of the enzyme topoisomerase II, reactivation of the suppressor protein p53, and induction of apoptosis by endoplasmic reticulum stress.^9,11–15^

Studies are being conducted to enhance the biological effects of naphthoquinones through a series of chemical modifications to their structure. In this study, we report on the synthesis of a novel series of derivative naphthoquinone compounds. Furthermore, we investigated its antitumor activity and molecular mechanisms in a series of *in vitro* and *in vivo* assays using OSCC cell lines and normal primary fibroblast cell models.

## 2 Results and Discussion

### 2.1 Chemistry

Among the natural 1,4-naphthoquinones, α-xyloidone **2** stands out, which is a nonabundant natural product but has several preparation methods. It is characterized by having a 1,4-naphthoquinonic ring fused with a chromenic heterocyclic ring.^16^ Typically, α-xyloidone preparation routes use lapachol as a starting reagent in several oxidative cyclization reactions. However, there are other methods that use lawsone and construct the chromenic ring. Highlighted are the single-step reactions from the Knoevenagel condensation between lawsone and α,β-unsaturated aldehydes, followed by an electrocyclization reaction,^17–19^ as highlighted in Scheme 1.

**Scheme 1:**
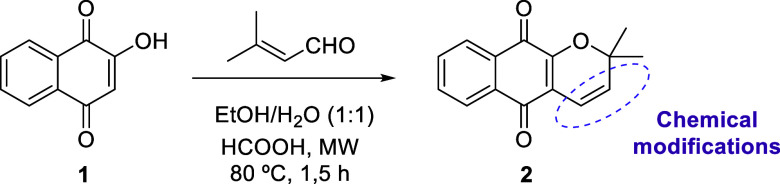
Synthesis of α-Xyloidone **2** from Lawsone **1**

In the literature, several 1,4-naphthoquinones containing arylthiols that have important biological activities are described.^20–22^ Likewise, there are 1,4-naphthoquinones with one or two arylthio groups linked to the olefin, which presented a wide range of biological activities derived from lawsone or juglone.^23,24^ For example, derivatives **3–6** containing arylthio groups showed potent antifungal activity, comparable to that of amphotericin B, against *C. tropicalis* (Figure 1).^25^

**Figure 1:**
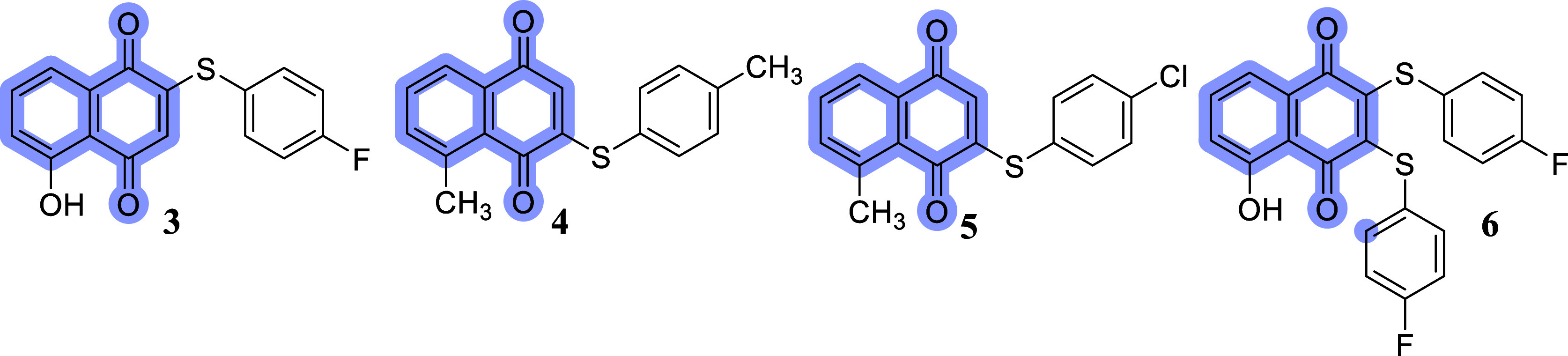
Examples of 1,4-naphthoquinones containing arylthiol groups.

Considering that the synthesis of 1,4-naphthoquinone is simple and can be obtained in high yield and, additionally, that the olefin is able to receive the insertion of arylthio groups leading to derivatives with potential biological activity, we decided to make modifications to the olefin of the chromene ring with several arylthio groups to obtain new 1,4-naphthoquinone derivatives.

With this objective in mind and as an additional extension of our study, the insertion of arylthiols into the double bond of the chromonic ring of α-xyloidone was evaluated. As far as we know, although it is of clear importance, this addition has not yet been studied. The synthesis of thionaphthoquinones (**7a–j**) derived from α-xyloidone (**2**) was carried out using the methodology presented in Scheme 2. α-Xyloidone (**2**) was prepared according to the literature previously reported by our research group.^19^ The reaction between α-xyloidone (**2**) and commercial thiols in ethanol under reflux conditions for 12 h led to the formation of the corresponding thionaphthoquinones (**7a–j**) with yields between 37 and 73% after filtration (Scheme 2).

**Scheme 2:**
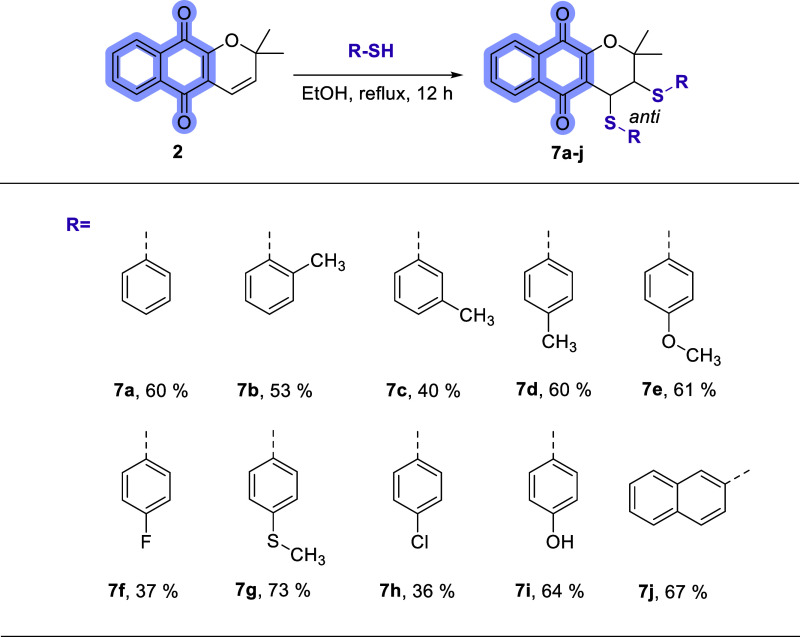
Synthesis of Dithioethers **7a–j** from α-Xyloidone **2**

Several spectroscopic methods, including FT-IR, ^1^H NMR, ^13^C NMR, and mass spectrometry, were used to characterize all of the thionaphthoquinones (**7a–j**). The ^1^H NMR of all of the desired hybrid molecules (**7a–j**) showed that the products were the result of a double insertion of the thiol into the unsaturation of α-xyloidone, due to the presence of duplicated signals in the aromatic hydrogen region of the thiol, in addition to the absence of methylene hydrogens expected for monoaddition, with two doublets integrating one hydrogen each being observed for all products, corresponding to the CH of the pyranic ring after the reaction. The formation of dithioethers **(7a–j)** can be explained by two sequential Thia–Michael reactions, made possible by the electronic conjugation present in the α-xyloidone structure. A plausible proposed mechanism for the formation of dithioethers (**7a–j**) is presented in Scheme 3. The anti-isomer was obtained for the dithioethers (**7a–j**), confirmed by the ^1^H-NOESY NMR spectrum (see Supporting Information—Figure S22). One representative dithioether **7d** was discussed here, and all other dithioethers demonstrated a similar profile. The ^1^H NMR spectra of compound **7d** was characterized under 500 MHz (δ, ppm) using DMSO-*d*_6_ as a solvent. The **7d** derivative had a total of 26 hydrogens: four aromatic hydrogens appeared in the range between 8.01 and 7.83, referring to the hydrogens of the naphthoquinonic system; eight aromatic hydrogens between 7.15 and 6.96 originated from the thiol; two hydrogens were exhibited in the CH region and showed chemical shifts at 4.20 and 3.97 as a doublet; and there were 6 methylic hydrogens of thiol (2.32 and 2.26) and 6 methylic hydrogens of the pyranic ring (1.74 and 1.66) as singlets. The ^13^C/APT NMR spectrum (125 MHz, DMSO-*d*_6_, δ, ppm) showed 20 signals, 9 in the even phase and 11 in the odd phase, with emphasis on the CH carbons at 62.55 and 55.55, corroborating the confirmation of the dithioether formed. The mass spectra of all dithioethers were determined and found to be in agreement with the expected values, confirming the successful synthesis of the intended derivatives.

**Scheme 3:**
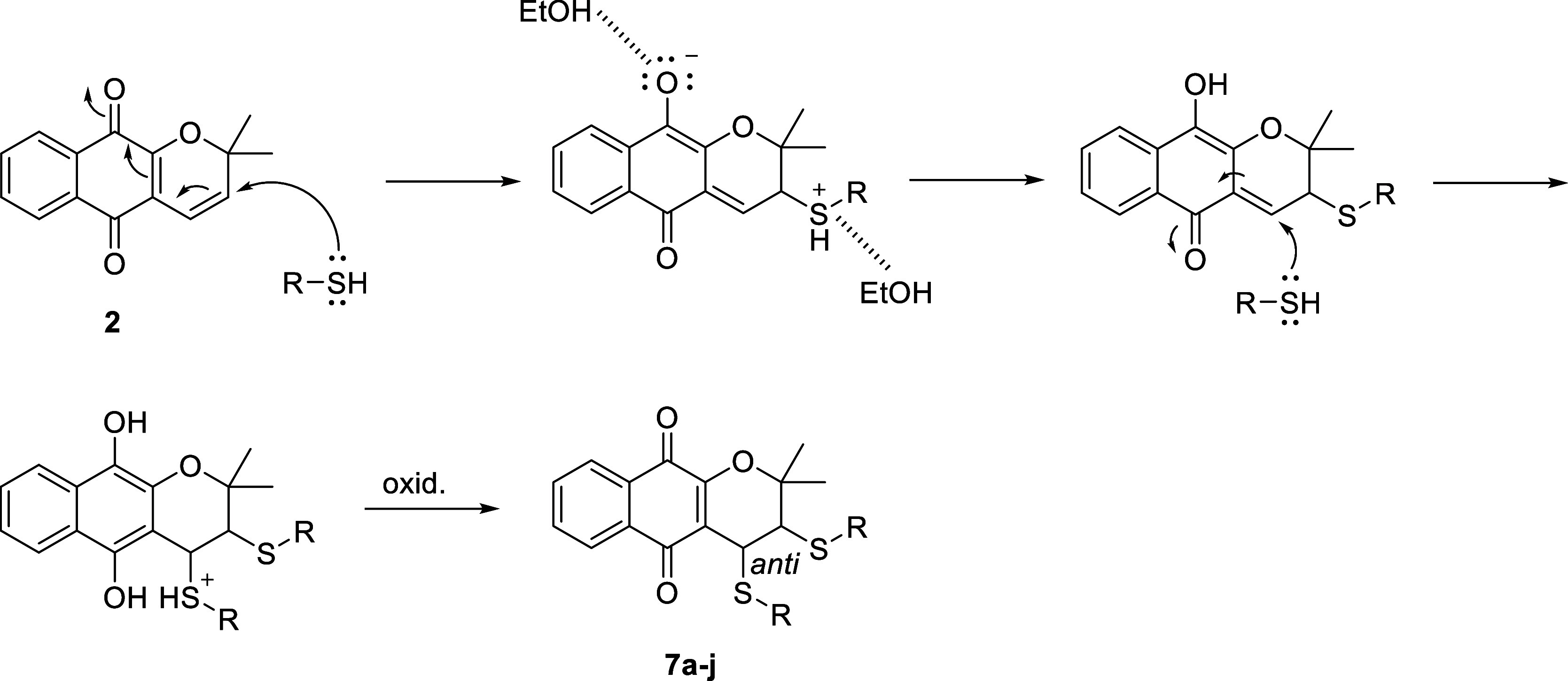
Plausible Mechanism for the Formation of Dithioethers **7a–j**

### 2.2 Biological Activity

#### 2.2.1 Thionaphthoquinone Derivatives **7a** and **7e** Show Cytotoxic Activity and Selectivity in Different *In Vitro* Cancer Models and Tolerability in Mice

Initially, the ten thionaphthoquinones (**7a–j**) were subjected to the MTT test. First, we screened oral cancer models, our main focus. For this, we started with the SCC-9 lineage since its a more sensitive lineage, and as controls, we used two chemotherapeutic agents, namely, carboplatin, the gold standard for the treatment of oral cancer,^26–28^ and shikonin, a naphthoquinone (NQ) with antitumor potential.^29^ Eight of the ten derivatives tested showed dose-dependent cytotoxicity and were subsequently tested on untransformed primary human gingival fibroblasts (Table 1). The anticancer activities of these molecules are reported here for the first time.

**Table 1:** Determination of IC_50_ of Thionaphthoquinone Compounds^a^

	**SCC-9—oral cancer**		**primary fibroblast**	**gingival**	**SI**
**compounds**	**IC_50_ (μM)**	**SD.**	**IC_50_ (μM)**	**SD.**	
**7a**	26.3	0.1	57.3	0.3	2.2
**7b**	34.8	0.2	50.9	0.8	1.5
**7c**	15.3	0.1	40.8	0.4	2.7
**7d**	30.9	0.1	60.1	0.5	1.9
**7e**	46.5	0.1	110.0	0.2	2.4
**7f**	35.3	0.2	40.0	0.6	1.1
**7g**	ND	ND	ND	ND	ND
**7h**	33.3	0.2	43.0	0.9	1.3
**7i**	17.2	0.3	32.6	0.3	1.9
**7j**	ND	ND	ND	ND	ND
Carboplatin	49.8	0.0	127.7	0.1	2.6
Shikonin	2.1	0.0	2.2	0.4	1.0

a SCC-9 cells (OSCC cells) were treated with the indicated compounds for 72 h, and cell viability was determined by the MTT assay. Results from at least 3 independent experiments. SD = standard deviation. ND = not determined.

The degree of selectivity of these derivatives can be expressed by their selective index (SI). An SI value ≥2 of a molecule represents selective toxicity to cancer cells, while an SI value <2 is considered generally toxic, so it can also cause cytotoxicity in normal cells.^30,31^ We observed that derivatives **7a** (SI 2.2), **7c** (SI 2.7), and **7e** (SI 2.4) were the most selective (Table 1, highlighted), being superior to the shikonin control (SI: 1.0) and similar to that of carboplatin (SI 2.6). As selectivity is one of the main factors that evaluate the effectiveness of chemotherapy agents, it is important to consider other oral cancer cell lines, which is why these three thionaphthoquinones (TNQs) were tested in two more OSCC tumor cell lines (SCC-4 and SCC-25). We found that among the three most selective derivatives, only 3c did not maintain an average selectivity index above 2, with an average SI result of 1.8 (Table 2). However, **7e** (SI = 4.2) surpassed those of carboplatin (SI = 3.5) and shikonin (SI = 2.0). On the other hand, **7a** (SI 3.2) obtained an average selectivity in the three lineages higher than shikonin and similar to that of carboplatin. With these results, only derivatives **7a** and **7e** were used for the following tests since they showed selectivity on average against all OSCC cell lines tested.

**Table 2:** Characterization of the Most Selective Thionaphthoquinone Compounds in Other OSCC Cells^a^

	**oral tumor cells**								
**compound**	**SCC-9**		**SCC-25**		**SCC-4**		**primary gingival fibroblast**		**average SI**
	**IC_50_**	**S.D.**	**IC_50_**	**S.D.**	**IC_50_**	**S.D.**	**IC_50_**	**S.D.**	
**7a**	26.3	0.1	28.2	0.2	10.6	0.1	57.3	0.3	3.2
**7c**	15.3	0.1	31.8	0.4	30.2	0.1	40.8	0.4	1.8
**7e**	46.5	0.1	13.0	0.2	57.1	0.2	110.0	0.2	4.3
carboplatin	49.8	0.0	38.3	0.1	27.7	0.1	127.7	0.1	3.5
shikonin	2.2	0.0	0.6	0.2	1.6	0.4	2.2	0.4	2.0

a The IC_50_ (μM) of three different OSCC cell lines (SCC-4, SCC-9, and SCC-25) for compounds **7a**, **7c**, and **7e** were calculated. SI = IC_50_ of the compound in normal oral fibroblast cells/IC_50_ of the same compound for each oral cancer cell line, and the average SI was found. All experiments are the results of at least three independent experiments. SD = standard deviation.

Our next screening was to evaluate the cytotoxic potential of the two TNQs in other *in vitro* cancer models (Table 3). For this, we used three tumor lines representative of colon (HCT-116), liver (HepG2), and melanocyte (B16-F10) cancers. Overall, TNQ **7e** showed SI similar to or higher than those of **7a** and carboplatin, being the most promising tested compound.

**Table 3:** SI of the Six Tumor Lines Tested with Thionaphthoquinones **7a** and **7e** and the Control

	**selective index (SI) per cell type**					
**compound**	**oral cancer**			**colon cancer**	**hepatocellular carcinoma**	**melanoma**
	**SCC-9**	**SCC-25**	**SCC-4**	**HCT-116**	**HepG2**	**B16-F10**
**7a**	2.2	2.0	5.4	2.7	2.1	1.4
**7e**	2.4	8.5	1.9	9.1	3.4	7.5
carboplatin	2.4	3.3	4.6	2.5	7.1	5.5

Based on this result, we evaluated the safety of using these two substances in biological systems. To rule out any surfactant activity that could lead to nonspecific cytotoxicity through damage to the cell membrane, we carried out hemolytic tests, where we verified that the two derivatives, **7a** and **7e**, did not demonstrate any surfactant property in the membranes. The test result (Figure 2A) shows less than 2% hemolysis at the highest concentration tested, 500 μM, more than 100 times higher than the IC_50_, when compared to the positive control, Triton X-100, which represents 100% of cell lysis in red blood cells. Further, carboplatin and the negative control (DMSO) had similar hemolytic capacity. The concentration at which they were tested represents more than ten times the respective IC_50_. Other experimental studies reported in the literature involving NQ derivatives show similar results.^32–34^ Together, these results indicate that both **7a** and **7e** are selective against nonhemolytic and oral cancer tumor cells, making *in vivo* testing possible.

**Figure 2:**
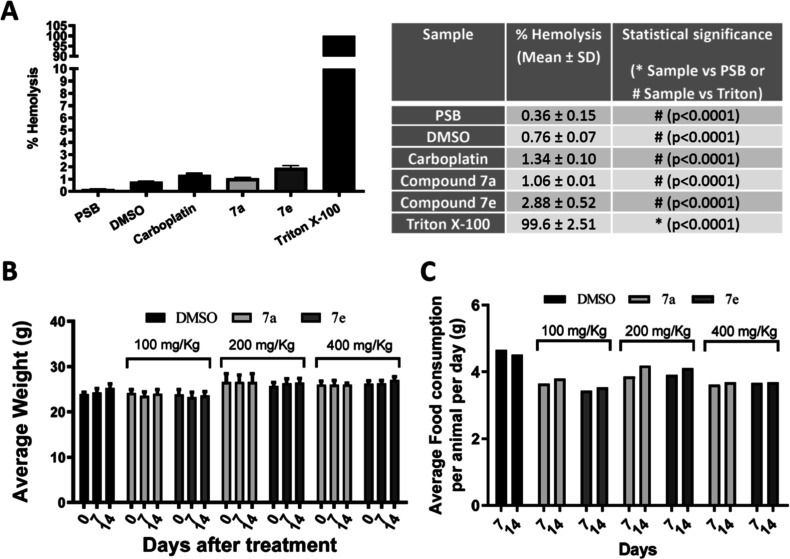
New TNQs are nonhemolytic and are tolerable in mice. (A) The hemolytic activity of compounds **7a** and **7e** was carried out at 500 μM. Left: graph; right: descriptive results after a one-way ANOVA with Dunnett’s posttest was performed, where all columns were significantly different from the control (Triton X) with *p* < 0.0001. (B,C) Acute toxicity study shows the average variation in body weight (B) and food consumption (C) of animals in the three treatment groups (100 mg/kg, 200 mg/kg, and 400 mg/kg) with 3 animals in each and the control group over 14 days.

In addition to evaluating safety and efficacy, preclinical animal testing also plays an important role in understanding the mechanisms of action of new molecules *in vivo*.^32,35^ To determine the tolerated dosage in animals, compounds **7a** and **7e** were subjected to acute toxicity testing in C56BL/6 mice to study their toxic potential. Three different groups of animals received a single intraperitoneal dose of 100, 200, and 400 mg/kg of derivatives **7a** and **7e** and were followed for 14 days. During the test, no morbidity and mortality were observed in animals treated with TNQ at any of the concentrations tested when compared to the DMSO control. Assessment of the abdominal cavity and macroscopic organs at necropsy indicated there were also no morphological changes or lesions in any of the tested groups when compared to the control group. Furthermore, there was no significant difference in body weight and food consumption compared to control animals at any dose (Figure 2B,C). According to the literature, other synthetic compounds based on NQ have already demonstrated this low toxicity in *in vivo* tests.^32^ ,^33^ ,^36^ Therefore, we did not find apparent limiting toxic effects in compounds **7a** and **7e** in mice at the concentrations tested, making these molecules promising candidates for further *in vivo* anticancer testing at higher doses.

#### 2.2.2 Thionaphthoquinones **7a** and **7e** Antitumoral Effects: Induction of Cell Death with Signs of Apoptosis and Autophagy and Induction of ROS Production and Antimigratory Capabilities

Given the results showing that compounds **7a** and **7e** are selective and well tolerated in mice, we next focused on determining the possible mechanism of cell death and the pathway involved. Chemotherapy is capable of inducing different types of cell death, and identifying the exact pathway is important in the development of new anticancer drugs.^37^ Observing the morphological changes in cells helps to characterize the type of cell death that is occurring. In apoptosis, cells shrink, produce membrane blebs, and release DNA fragments and apoptotic bodies. Through the timelapse microscopy (Figure 3A), OSCC cells treated with the compounds showed the formation of membrane blebs at early times, between 16 and 48 h, followed by cell shrinkage, both suggestive of apoptosis (Video S1). To further investigate the cytotoxic process and exclude other cell pathways, we tested the possibility of caspase-induced cell death. Apoptosis depends on an intracellular proteolytic cascade mediated by caspases, which is the main biological characteristic of apoptosis. Caspases 3, 6, and 7 are responsible for the degradation of cellular proteins and for the fragmentation of the nucleus (DNA) and the cytoskeleton, leading to the disassembly of the cell, actively participating in apoptosis.^38,39^ In Figure 3B, we observed that in the cells treated with **7a** and **7e**, they demonstrate signs of the formation of membrane bubbles and cell retraction detectable by the presence of several cells in rounded and fusiform shapes with positive staining for active effector caspases 3 and 7 (71 and 78.6%, respectively), while the negative control (DMSO) demonstrated only 3.6% of positive cells (Figure 3C). Both morphological and biochemical data support the induction of the apoptotic pathway.

**Figure 3:**
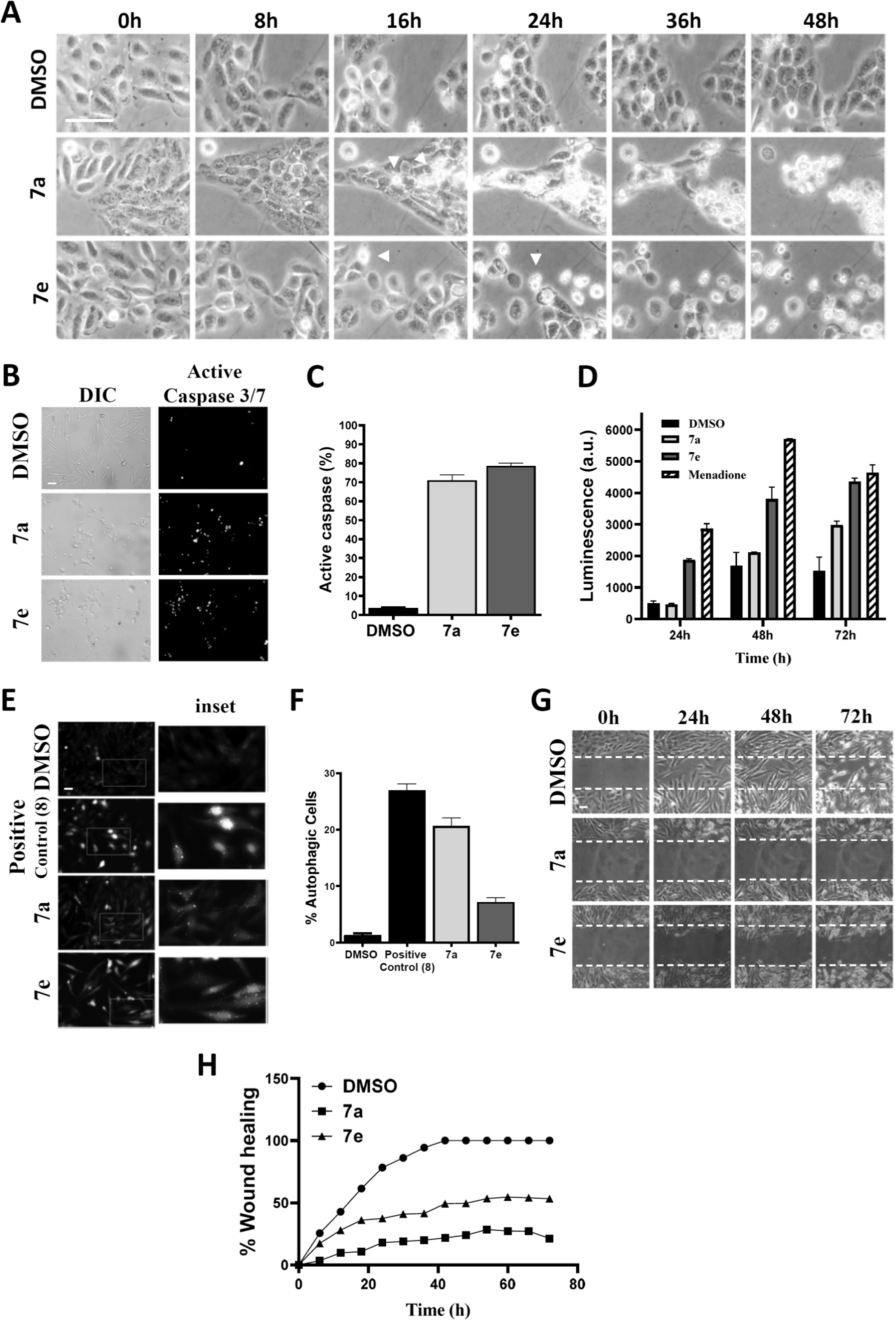
Cell death mechanism investigation for the most selective naphthoquinone derivatives **7a** and **7e** in OSCC cells. All experiments were performed using SCC-9 cells. (A) Timelapse video microscopy images obtained from Video S1 reveal that **7a** and **7e** (2 × IC_50_) induce the appearance of membrane blebs, the loss of membrane integrity, and cellular rupture, which were intensified at 36 and 48 h. (B) Representative microscopic images of SCC-9 cells treated for 24 h with (2 × IC_50_) **7a** or **7e** and DMSO demonstrating cell morphology and staining for active caspase 3/7. (A,B) Scale bar is 100 μm. (C) Graph quantifying SCC-9 cells with active caspase 3/7 in relation to the total cell number. (D) SCC-9 cells were treated with the **7a** or **7e** partition for 24, 48, and 72 h before ROS production was measured, with normalized data considering menadione (positive control) as 100% of ROS production. (E) Determination of autophagosome formation. SCC-9 expressing LC3 fused to GFP protein were treated with 1 × IC_50_ of **7a**, **7e**, and DMSO, or as a positive control, we used a coumarin–naphthoquinone hybrid, **8**, known to induce autophagy in our model^40^ for 48 h, and puncta formation was observed by fluorescent microscopy. The scale bar is 100 μm. (F) Quantification of autophagosome formation (puncta) was done through the ratio between the number of cells with autophagosome formation and the total number of cells per field. (G) Wound healing assay. Timelapse video microscopy images were obtained from Video S2 and are presented in a representative time sequence of DIC images in timelapse observation at 20× magnification. Cells were treated with sublethal doses (1/8 of IC_50_) of **7a** (1.64 μM) and **7e** (2.91 μM). To avoid further cell proliferation, 1 μL/mL of mitomycin c was used at a concentration of 0.5 mg/mL. The scale bar is 100 μm. (H) Graph representing the rate (%) and time (h) of wound closure. All results are from at least 3 independent experiments.

Reactive oxidative species (ROS) research has been widely used to create new anticancer drugs, since ROS can induce damage to DNA, lipids, and cellular proteins, contributing to the death of neoplastic cells.^41,42^ Furthermore, it is well established in the literature that different NQs can induce ROS, being one of the properties that most confers antineoplastic and apoptosis-inducing action to this class of substances.^43,44^ Our results show that **7a** and **7e** both induced the generation of ROS in SCC-9 cells, despite lower production than the positive control (menadione), but more significant than the solvent alone (Figure 3D). The result obtained by derivative **7e** is worth highlighting, where the production of ROS, within 72 h, approached the result of the positive control. Further, ROS production can induce apoptosis and also induce autophagy, leading to cell death, probably upon exposure to modest doses of H_2_O_2_.^45–47^ Therefore, we decided to perform a molecular evaluation of autophagic labeling to confirm our findings. For this, we used SCC-9 cells that express the microtubule-associated protein 1A/1B-light chain 3 (LC3) fused with the GFP protein. LC3 is a protein that, during autophagy, is recruited to autophagosomes that can be visualized as dots under microscopy, and its aggregation in puncta is implicated in autophagy.^9,48^ As seen in Figure 3E and quantified in Figure 3F, **7a** induced LC3-positive puncta indicative of autophagy in 20.5% of cells and **7e** in 7.4%, whereas the negative control had only about 1%. The result of **7a** was close to that of the positive control (compound **8**, Figure S1), an already described naphthoquinone-triazole-coumarin hybrid, able to induce autophagy,^40^ which induced autophagy in 26.5% of the cells, thus indicating that autophagy is also present in the death process induced by the two molecules, most notably in **7a**.

Last but not least, we investigated the antimigratory capacity of molecules **7a** and **7e**. Cell migration plays a crucial role in several normal physiological processes.^49,50^ However, this same process is also frequently exploited by tumor cells to promote the invasion and spread of cancer to other tissues and organs in the human body.^51–53^ In Figure 3G and Video S2, we observe the results of the wound healing assay on a monolayer of SCC-9 cell lines. Control cells demonstrate rapid progression in cell layer migration, reaching 100% closure at around 40 h. On the other hand, when treated with sublethal concentrations (1/8 of the determined IC_50_), both **7a** and **7e** demonstrate the ability to inhibit cell migration. Derivative **7a** showed a gradual rate of wound closure, reaching a peak of 28.4% closure in 72 h, while substance **7e** reached 54.7% in 72 h (Figure 3H). We previously showed that another naphthoquinone Mannich base derived from lawsone was unable to reduce wound healing, suggesting a special skill to these two novel naphthoquinone-based compounds.^54^ Overall, compounds **7a** and **7e** significantly delayed cell migration, suggesting that both may have an important role in the treatment of oral cancer, inhibiting the migration of tumor cells and potentially reducing the risk of invasion and thus metastases.

#### 2.2.3 TNQs **7a** and **7e** Have Promising Prospects in *In Silico* Assessments of Physical Chemistry and Pharmacokinetics

The analysis of the physicochemical properties of new compounds allows a rapid and economical initial screening of the potential of these drug candidate molecules. To this end, *in silico* physicochemical predictions play an important role in these evaluations.^55,56^ Therefore, a set of relevant chemical and biological properties of compounds **7a** and **7e** was calculated and compared with controls used in the clinic (carboplatin and doxorubicin) using the SwissADME pharmacokinetic prediction server. Lipinski’s “rule of 5” was used to evaluate oral bioavailability according to four parameters: (1) the logarithm of the octanol/water partition coefficient (LogP ≤5 or MLogP ≤4.15); (2) the number of hydrogen bond acceptors (nON ≤10); (3) the number of hydrogen bond donors (nOH/NH ≤5); and (4) molecular weight (MW ≤500 Da). Compounds with two or more violations of these criteria probably do not exhibit good permeation and absorption.^55,57^ Compounds **7a** and **7e** each had one violation of Lipinski’s “rule of 5”, while the controller drugs doxorubicin and carboplatin had three and no violations, respectively (Table 4).

**Table 4:** Physicochemical Descriptors of Compounds **7a** and **7e** and the Control Chemotherapy Drugs Doxorubicin and Carboplatin

**compounds**	MLogP*	**nON**	**nOH/NH**	**MW**	**Lipinski’s violations** ^a^	**TPSA (Å^2^)**
**7a**	4.15	3	0	458.59	1	93.97
**7e**	3.4	5	0	518.64	1	112.43
doxorubicin	–2.1	12	6	543.52	3	206.07
carboplatin	–1.79	6	4	371.25	0	126.64

a Number of violations to the Lipinski’s “rule of 5”: MLogP <4.15; MW, molecular weight ≤500; nON, number of hydrogen bond acceptors ≤10; and nOH/NH, number of hydrogen bond donors ≤5.

Furthermore, topological polar surface area (TPSA) is one of the parameters used to predict drug cell permeability, oral bioavailability, and intestinal absorption.^58^ Compounds with TPSA above 140 Å^2^ show low membrane permeability, while compounds with TPSA below 60 Å^2^ show high permeability and human intestinal absorption.^58^ The values in Table 4 show that both **7a** (93.97 Å^2^) and **7e** (112.43 Å^2^) have values within the permeability range for TPSA. On the other hand, carboplatin (126.6 Å^2^) and doxorubicin (206.1 Å^2^) presented higher TPSA values, indicating that these compounds probably have low cellular permeability and intestinal absorption.

The vast majority of drugs are developed in such a way as to allow their administration via the oral route, which is by far the most convenient route. It brings great advantages in terms of patient adherence to treatment; its cost-benefit is good; and it is easy to produce on a large scale in large quantities.^59^ Thus, pharmacokinetic assessments on absorption become of great importance in the first phase of any candidate substance for an oral drug.^60^ Therefore, we use *in silico* tools that use computational predictive methods ADME/T (Absorption, Distribution, Metabolism, Excretion, and Toxicity) through different free access providers on the web to try to eliminate any bias existing in individual servers.^61–64^ Not all ADMET servers make every prediction analyzed, and we discuss mostly the consensus between them. Reinforcing the absorption and permeability prediction based on RO5, the QSAR-based method available on the admetSAR 2.0 also predicted an oral bioavailability of compound **7a**, while **7e** and the controls doxorubicin and carboplatin were predicted to exhibit poor oral bioavailability (Table 5). In fact, experimental studies have demonstrated the low oral bioavailability of these drugs,^65,66^ supporting the reliability of our predictions, which, in turn, support that compound **7a** is suitable for oral administration, unlike the evaluated anticancer drugs and compound **7e**.

**Table 5:** Predicted Pharmacokinetic Properties (ADME) of Compounds **7a** and **7e** and the Chemotherapeutic Agents, Carboplatin and Doxorubicin, Using Four Different Computational Tools (admetSAR 2.0, ADMETlab 2.0, pkCSM, and SwissADME)^a^

**ADME**	**human oral bioavailability**				*P*-glycoprotein inhibitor				*P*-glycoprotein substrate			
Web servers	**7a**	**7e**	Carbo	Dox	**7a**	**7e**	**Carbo**	**Dox**	**7a**	**7e**	Carbo	**Dox**
admetSAR	+0.54	–0.50	–0.60	–0.91	+0.77	+0.91	–0.92	–0.99	–0.93	–0.90	–0.99	–0,99
ADMETlab 2.0	ND	ND	ND	ND	+0.99	+1.0	–0.00	+0.86	–0.00	–0.00	–0.01	+1.0
pkCSM	ND	ND	ND	ND	+	+	–	+	–	–	–	+
SwissADME	ND	ND	ND	ND	ND	ND	ND	ND	–	+	–	+

a ND: non-determined; (+) yes; (−) no.

Phosphoglycoprotein-P (Pg-P) is a glycosylated membrane protein belonging to the family of ABC transporters,^67^ encoded by the ABCB1 gene.^68^ It is associated with multidrug resistance (MDR), which poses the primary challenge in cancer treatment via chemotherapy.^68^ Hence, we assessed whether TNQs **7a** and **7e** could function as substrates or inhibitors of this protein. Both were predicted to be Pg-P inhibitors but not substrates in three of the four analyzed databases (Table 5). This is promising, as the prediction of Pg-P inhibitors implies their potential to decrease the activity of this protein, thereby enhancing the concentration of specific drugs in target tissues, which could augment their therapeutic efficacy. Furthermore, Pg-P inhibitors may assist in overcoming this resistance, restoring tumor sensitivity to drugs, and potentially serving as adjuvants for other medications.^67,69–71^ On the contrary, there was no indication that carboplatin acted as a substrate or inhibitor of Pg-P. Further, doxorubicin was identified as a substrate by three servers and an inhibitor by two servers of this protein, suggesting its transportation by Pg-P and expulsion through these effluxes. These predictions align with available experimental data for both control drugs.^72^ Consequently, the discovery of new molecules with Pg-P inhibition capacity capable of reversing MDR in cancer cells represents an intriguing avenue for improving chemotherapy.

Based on the results of toxicology predictions for molecules **7a** and **7e**, in comparison to the positive controls carboplatin and doxorubicin, several important trends were observed (Table 6). Concerning carcinogenicity, carboplatin received exclusively negative predictions, indicating a low risk of causing cancer. Conversely, doxorubicin and compounds **7a** and **7e** exhibited mixed predictions, with predominantly negative results and a single positive prediction, all from ADMETlab 2.0. This suggests uncertainty regarding the carcinogenic potential of these molecules, necessitating further detailed assessment in additional studies. However, it is noteworthy that doxorubicin, while effective in treating cancer, can have carcinogenic effects, especially with prolonged or high-dose use, underscoring the importance of utilizing multiple servers for a more comprehensive analysis.

**Table 6:** *In Silico* Predicted Toxicological Properties of Compounds **7a** and **7e** and the Chemotherapeutics Carboplatin and Doxorubicin Using Five Different Computational Tools (admetSAR 2.0, ADMETlab 2.0, Osiris, pkCSM, and SwissADME)^a^

**toxicology**												
**parameters**	**carcinogenicity**		**cardiotoxicity**				**hepatotoxicity**					
**substance**	**Carb**	**Dox**	**7a**	**7e**	**Carb**	**Dox**	**7a**	**7e**	**Carb**	**Dox**	**7a**	**7e**
admetSAR	–0.76	–0.93	–0.97	–0.96	ND	ND	ND	ND	+0.68	–0.85	+0.68	+0.85
ADMETlab 2.0	–0.49	+0.92	+0.82	+0.91	ND	ND	ND	ND	+0.83	–0.46	+0.98	+0.99
Osiris	–1	–1	–1	–1	ND	ND	ND	ND	ND	ND	ND	ND
pkCSM	ND	ND	ND	ND	ND	ND	ND	ND	–	–	–	–
PROTOX 3.0	–0.80	–0.90	–0.57	–0.57	–0.60	+0.64	–0.79	–0.72	–0.69	–0.86	–0.58	–0.58

a ND: non-determined; (+) yes; (−) no.

In terms of cardiotoxicity, only PROTOX 3.0 returned a result where compounds **7a**, **7e**, and carboplatin yielded negative predictions, while doxorubicin was positive. This indicates a potential elevated risk of cardiac damage associated with doxorubicin, a finding consistent with clinical observations and literature reports.^73,74^

Regarding hepatotoxicity, doxorubicin primarily received negative predictions, indicating a low risk of liver toxicity. Carboplatin and the new TNQs yielded mixed predictions comprising both negative and positive outcomes. However, while myelosuppression is the dose-limiting side effect of carboplatin, other side effects, including hepatotoxicity, have been reported in the literature.^75,76^ Similarly, doxorubicin has been associated with liver toxicity in the literature.^77^ This suggests potential for liver toxicity with these molecules, necessitating further investigation.

When investigating the mean lethal dose (LD_50_), we obtained a prediction from the PROTOX ∼ 3.0 server for the two TNQs, 2000 mg/kg, while for carboplatin, it was 343.0 mg/kg, and for doxorubicin, 205 mg/kg. These data suggest the low toxicity of the two TNQs in relation to controls, which was confirmed in the *in vivo* acute toxicity test, where we did not observe mortality or lethality among mice in all tested concentrations.

In summary, the results of these predictions highlight the importance of performing a comprehensive assessment of the toxicological risks associated with novel **7a** and **7e** molecules before they advance to preclinical and clinical studies. Although initial predictions may provide useful insights, they must be interpreted with caution and confirmed through laboratory experiments and additional toxicity studies. This approach is essential to guarantee the safety and efficacy of these molecules as potential candidates for antineoplastic drugs.

Next, we continued the investigation of possible mechanisms of cell death involved in the action of TNQ **7e**, as it presents a better cytotoxic and selective profile than that of **7a** in the initial screening of cytotoxicity and selectivity in different cancer models. Our next study was to perform reverse molecular docking of this compound.

#### 2.2.4 Analysis of Putative Antitumor Targets of Compound **7e** by *In Silico* Reverse Docking

We employed a molecular modeling approach to identify the potential molecular target of the most active compound **7e**. A group comprising six proteins known as anticancer targets of naphthoquinone derivatives were selected as possible targets of the evaluated compound. Based on our predictions, the 3S,4S enantiomer likely plays a significant role in the anticancer activity of compound **7e**; therefore, the results presented refer specifically to the docking of this enantiomer.

Some of the targets evaluated were PKM2, which acts as a metabolic regulator in tumor cells,^78^ and RSK2, which is a serine/threonine kinase that plays a regulatory role in various cellular processes such as proliferation, cell cycle progression, and apoptosis.^79,80^ These targets were evaluated since both proteins are important therapeutic targets in many types of cancer and are inhibited by quinone derivatives such as lapachol and shikonin.^81–84^ Our results revealed that compound **7e** displays a very different binding mode compared with the known inhibitor shikonin with PKM2 (data not shown), suggesting that this enzyme is not the target of this compound. By contrast, compound **7e** bound to RSK2 similar to the cocrystallized inhibitor 2NS and lapachol, which inhibits this protein and induces intrinsic apoptosis in esophageal squamous carcinoma cells with an IC_50_ of 2 μM.^80^ Compound **7e**, as well as lapachol, was involved in π-sigma interactions with L74 and L200, while the same interaction with L74 was observed for the ligand 2NS (Figure 4A). In addition to these similar interactions, compound **7e** exhibited similar binding affinity with this protein (−7.1 kcal/mol) in comparison to lapachol and ligand 2NS (−8.1 and −10.3 kcal/mol, respectively).

**Figure 4:**
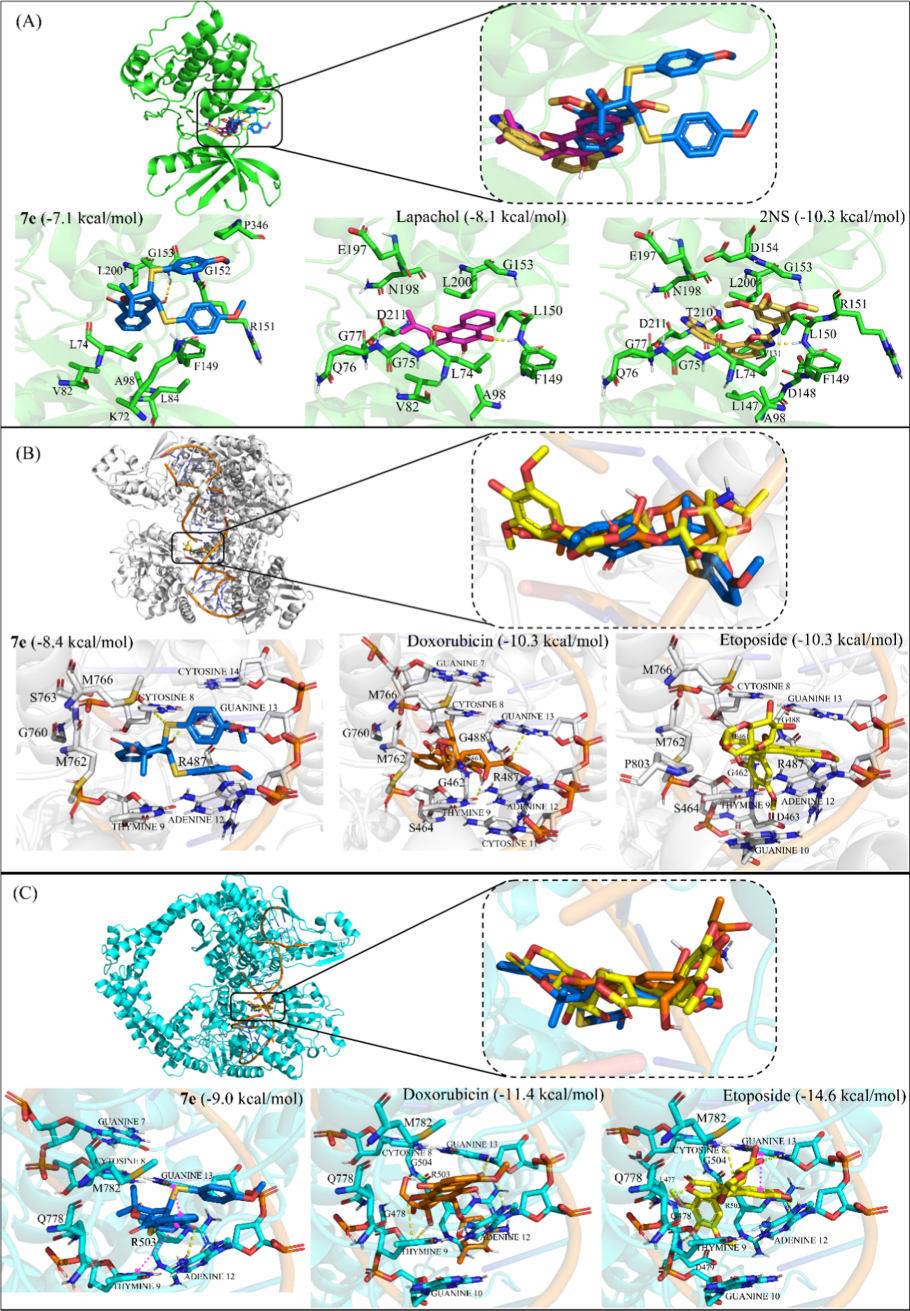
Molecular docking of the compound (A) **7e** (−7.1 kcal mol^–1^) with the RSK2 and comparison with the docking of known inhibitor lapachol (−8.1 kcal mol^–1^) and cocrystallized inhibitor 2NS (−10.3 kcal mol^–1^); (B) **7e** (−8.4 kcal mol^–1^) with the topoisomerase IIα-DNA and comparison with the docking of known inhibitor doxorubicin (−10.3 kcal mol^–1^) and cocrystallized inhibitor etoposide (−10.3 kcal mol^–1^); and (C) **7e** (−9 kcal mol^–1^) with the topoisomerase IIβ-DNA and comparison with the docking of known inhibitor doxorubicin (−11.4 kcal mol^–1^) and cocrystallized inhibitor etoposide (−14.6 kcal mol^–1^). Hydrogen bonds are shown as yellow dashed lines, and π–π-stacked interactions are shown as pink dashed lines.

Furthermore, we evaluated the binding mode of compound **7e** with the DNA-binding domain of topoisomerases I, IIα, and IIβ and compared it to the cocrystallized ligands topotecan and etoposide as well as the anticancer drug doxorubicin. Doxorubicin is an anticancer drug used clinically in the treatment of various neoplasms; it is known to inhibit DNA topoisomerases through intercalation with DNA, as well as some quinones.^85–87^ Although **7e** has a polycyclic moiety like topotecan, it did not intercalate into the nucleobases of DNA bound to topoisomerase I (data not shown).

On the other hand, this compound exhibited a binding mode comparable to the cocrystallized ligand etoposide within the DNA structure bound to topoisomerases IIα and IIβ. Yet, compound **7e** showed a similar binding energy (−8.4 kcal/mol) compared to doxorubicin (−10.3 kcal/mol) with topoisomerase IIα (Figure 4B). Regarding this enzyme, compounds **7e** and doxorubicin present an overlap in their structures; therefore, they have a similar interaction profile, especially in the naphthoquinone moiety. Furthermore, π-sulfur interactions were observed involving the sulfur atoms bonded to the 3S and 4S carbons.

Within topoisomerase IIβ, compound **7e** was superimposed on doxorubicin and the cocrystallized ligand etoposide. In topoisomerase IIβ, an overlap of compounds **7e** and doxorubicin is also observed. Furthermore, in compound **7e**, the substituent containing sulfur bonded to the 3S carbon shows an interesting interaction profile, since π–π-stacked interactions are observed with guanine-13, which stabilize the compound in DNA and may facilitate intercalation between the bases, as observed for the cocrystallized ligand etoposide (Figure 4C). Finally, the binding affinity obtained for compound **7e** was like those obtained for doxorubicin and etoposide (−9.0 kcal/mol, −11.4 kcal/mol, and −14.6 kcal/mol, respectively).

Thus, given the comparable binding mode and shared interactions with known inhibitors of RSK2, topoisomerase IIα, and topoisomerase IIβ, our findings suggest that compound **7e** may target these proteins to trigger its anticancer activity. However, further experiments are needed to validate these putative targets, including hTopoIIα-DNA cleavage complex formation assays and enzymatic inhibition studies of RSK2.

## 3 Conclusion

In summary, ten novel compounds derived from α-xyloidone and commercial thiols were synthesized via the thia-Michael addition reaction, and their antitumor potential was evaluated in OSCC cells. Among these, compounds **7a** and **7e** demonstrated potent cytotoxicity (IC_50_ of 26.3 and 46.5 μM, respectively) and favorable selectivity indices (3.2 and 4.2), with efficacies comparable to or exceeding those of standard chemotherapy agents. Both derivatives effectively inhibited tumor cell migration and exhibited no toxicity in acute animal studies, being well tolerated at all tested doses. Our data further indicate that these thionaphthoquinones induce cell death via apoptosis and autophagy. Molecular docking analyses, performed for compound **7e** due to its relatively higher cytotoxicity, suggest that its anticancer activity may be mediated by targeting key proteins, such as RSK2, topoisomerase IIα, and topoisomerase IIβ, but further experiments are required to confirm these interactions and elucidate the precise molecular mechanisms involved. Importantly, both compounds display pharmacological profiles within desirable parameters for drug development, underscoring their promise for future preclinical trials.

## 4 Materials and Methods

### 4.1 Chemistry

#### 4.1.1 Materials and Methods

IR spectra were obtained via a PerkinElmer model 1420 double-beam spectrometer. ^1^H NMR and ^13^C NMR spectra were obtained via a Varian VNMRS 500 MHz or Varian VNMRS 300 MHz or Bruker Ascend 500 MHz NMR spectrometer in CDCl_3_ or DMSO-*d*_6_ using TMS as the internal reference. Chemical shifts are reported in ppm (δ), and coupling constants (*J*) are reported in Hertz. High-resolution mass spectra were obtained on Micromass-Q-TOF (Waters) in the ESI mode (HR-ESI-MS). Analytical-grade solvents were used. TLC was performed on Silica Gel 60-F-254 (ref Merck 5554), and spots were visualized by irradiation with UV light (254 and 365 nm). Column chromatography was performed with silica gel 60 (230–400 mesh ATSM, Acros Organics). Melting points were obtained on a Fisher Johns apparatus and are reported as uncorrected values. Compound **2** was prepared using an Anton Paar monowave 300 microwave reactor.

#### 4.1.2 General Procedure for Preparing α-Xyloidone **2**

α-Xyloidone was synthesized according to the methodology previously described by our research group.^19^ A 10 mL microwave tube was loaded with **1** (11.5 mmol), 3-methyl-2-butenal (11.5 mmol), formic acid (17.3 mmol), and 5 mL of a 1:1 ethanol/water mixture (v/v), and the resulting mixture was irradiated for 1.5 h. The internal temperature reached 80 °C. The solvent was evaporated under reduced pressure, and the crude mixture was extracted with ethyl acetate (30 mL). The organic layer was washed with water (3 × 20 mL), dried over anhydrous sodium sulfate, filtered, and concentrated under reduced pressure. The residual solid product was purified by column chromatography using silica gel and a gradient of hexane/EtOAc as the eluent. The α-xyloidine was obtained in 91% yield as an orange solid.

#### 4.1.3 2,2-Dimethyl-2*H*-benzo­[*g*]­chromene-5,10-dione (**2**)

^1^H NMR (CDCl_3_, 500 MHz) δ: 8.09 (dd, *J* = 7.1, 1.9 Hz, 2H), 7.71 (td, *J* = 7.1, 1.4 Hz, 1H), 7.68 (td, *J* = 7.1, 1.4 Hz, 1H), 6.65 (d, *J* = 10.0 Hz, 1H), 5.72 (d, *J* = 10.0 Hz, 1H), 1.55 (s, 6H). ^13^C NMR (CDCl_3_, 125 MHz) δ: 181.98, 179.97, 152.56, 134.08, 133.32, 131.70, 131.63, 131.00, 126.35, 117.98, 115.60, 80.56, 28.51.

#### 4.1.4 General Procedures for the Synthesis of **7a–7j**

In a round-bottom flask, α-xyloidone (0.75 mmol) was added, and the mixture was diluted in 10 mL of ethanol. Then, 3.3 mmol of the respective thiol was added, and the resulting mixture reaction was refluxed for 12 h. After, the mixture was cooled to room temperature, leading to the formation of a precipitate. The solid was collected by simple filtration, washed with hexane, and dried at room temperature, obtaining the properly substituted thionaphthoquinones as solids with yields ranging from 36 to 73%.

#### 4.1.5 2,2-Dimethyl-3,4-bis­(phenylthio)-3,4-dihydro-2*H*-benzo­[*g*]­chromene-5,10-dione (**7a**)

**7a** was obtained as a yellow powder with 60% yield. mp 183.7–183.9 °C. ^1^H NMR (DMSO-*d*_6_, 500 MHz) δ: 8.02–8.01 (m, 1H), 8.01–7.99 (m, 1H), 7.87 (td, *J* = 7.4 e 1.6 Hz, 1H), 7.83 (td, *J* = 7.4 e 1.6 Hz, 1H), 7.29–7.18 (m, 10H), 4.32 (d, *J* = 2.2 Hz, 1H), 4.11 (d, *J* = 2.2 Hz, 1H), 1.74 (s, 3H), 1.66 (s, 3H). ^13^C NMR (DMSO-*d*_6_, 75 MHz) δ: 182.67, 178.96, 153.74, 135.65, 135.10, 134.16, 132.93, 132.77, 132.19, 131.90, 131.07, 129.79, 129.57, 128.32, 128.01, 126.48, 126.28, 118.81, 81.00, 55.93, 45.43, 27.56, 27.53. HRMS (ESI^+^) *m*/*z*: calcd for C_27_H_22_NaO_3_S_2_^+^, 481.0908. Found, 481.0918 [M + Na]^+^.

#### 4.1.6 2,2-Dimethyl-3,4-bis­(*o*-tolylthio)-3,4-dihydro-2*H*-benzo­[*g*]­chromene-5,10-dione (**7b**)

**7b** was obtained as a yellow powder with 53% yield. mp 194.9–195.3 °C. ^1^H NMR (DMSO-*d*_6_, 500 MHz) δ: 8.05–8.03 (m, 1H), 8.02–8.00 (m, 1H), 7.88 (td, *J* = 7.5 e 1.8 Hz, 1H), 7.84 (td, *J* = 7.5 e 1.8 Hz, 1H), 7.24 (td, *J* = 7.4 e 1.3 Hz, 1H) 7.20–7.15 (m, 4H), 7.06 (d, *J* = 7.8 Hz, 1H), 7.01–6.94 (m, 2H), 4.29 (d, *J* = 1.0 Hz, 1H), 3.88 (d, *J* = 1.7 Hz, 1H), 2.24 (s, 3H), 2.14 (s, 3H), 1.80 (s, 3H), 1.79 (s, 3H). ^13^C NMR (DMSO-*d*_6_, 125 MHz) δ: 182.06, 178.38, 153.44, 140.71, 139.41, 134.24, 134.03, 133.49, 133.45, 131.92, 131.62, 130.56, 130.51, 130.21, 128.37, 127.82, 127.48, 126.71, 126.45, 125.64, 117.81, 80.29, 53.83, 43.29, 27.47, 27.39, 19.92. HRMS (ESI^+^) *m*/*z*: calcd for C_29_H_26_NaO_3_S_2_^+^, 509.1221. Found, 509.1226 [M + Na]^+^.

#### 4.1.7 2,2-Dimethyl-3,4-bis­(*m*-tolylthio)-3,4-dihydro-2*H*-benzo­[*g*]­chromene-5,10-dione (**7c**)

**7c** was obtained as a yellow powder with 41% yield. mp 137.8–138.2 °C. ^1^H NMR (DMSO-*d*_6_, 500 MHz) δ: 8.02 (t, *J* = 1.7 Hz, 1H), 8.00 (t, *J* = 1.7 Hz, 1H), 7.87 (td, *J* = 7.5, 1.6 Hz, 1H), 7.83 (td, *J* = 7.4, 1.5 Hz, 1H), 7.13–7.03 (m, 8H), 4.35 (d, *J* = 2.3 Hz, 1H), 4.03 (d, *J* = 2.3 Hz, 1H), 2.20 (s, 3H), 2.15 (s, 3H), 1.74 (s, 3H), 1.68 (s, 3H). ^13^C NMR (DMSO-*d*_6_, 125 MHz) δ: 182.06, 178.39, 153.27, 138.66, 138.30, 134.24, 134.51, 133.55, 132.96, 131.97, 131.70, 130.50, 129.53, 128.85, 128.66, 128.61, 128.48, 128.30, 125.86, 125.72, 118.51, 80.58, 55.44, 44.85, 27.13, 26.83, 20.68, 20.55. HRMS (ESI^+^) *m*/*z*: calcd for C_29_H_26_NaO_3_S_2_^+^, 509.1221. Found, 509.1236 [M + Na]^+^.

#### 4.1.8 2,2-Dimethyl-3,4-bis­(*p*-tolylthio)-3,4-dihydro-2*H*-benzo­[*g*]­chromene-5,10-dione (**7d**)

**7d** was obtained as a yellow powder with 60% yield. mp 182.1–182.4 °C. ^1^H NMR (DMSO-*d*_6_, 500 MHz) δ: 8.01 (t, *J* = 1.7 Hz, 1H), 8.00 (t, *J* = 1.7 Hz, 1H), 7.87 (td, *J* = 7.5, 1.5 Hz, 1H), 7.83 (td, *J* = 7.4, 1.5 Hz, 1H), 7.15 (d, *J* = 8.1 Hz, 2H), 7.11 (d, *J* = 8.1 Hz, 2H), 7.02 (d, *J* = 7.9 Hz, 2H), 6.96 (d, *J* = 7.8 Hz, 2H), 4.20 (d, *J* = 2.0 Hz, 1H), 3.97 (d, *J* = 2.1 Hz, 1H), 2.32 (s, 3H), 2.26 (s, 3H), 1.74 (s, 3H), 1.66 (s, 3H). ^13^C NMR (DMSO-*d*_6_, 125 MHz) δ: 181.95, 178.29, 153.08, 137.59, 137.18, 134.38, 133.43, 132.95, 132.09, 131.62, 131.25, 130.44, 129.63, 129.42, 128.29, 125.78, 125.61, 118.23, 80.27, 55.56, 45.28, 27.03, 20.51, 20.42. HRMS (ESI^+^) *m*/*z*: calcd for C_29_H_26_NaO_3_S_2_^+^, 509.1221. Found, 509.1222­[M + Na]^+^.

#### 4.1.9 3,4-Bis­((4-methoxyphenyl)­thio)-2,2-dimethyl-3,4-dihydro-2*H*-benzo­[*g*]­chromene-5,10-dione (**7e**)

**7e** was obtained as a yellow powder with 61% yield. mp 168.3–168.8 °C. ^1^H NMR (DMSO-*d*_6_, 500 MHz) δ: 8.03–8.00 (m, 1H), 8.00–7.99 (m, 1H), 7.88 (td, *J* = 7.5, 1.5 Hz, 1H), 7.83 (td, *J* = 7.5, 1.4 Hz, 1H), 7.23 (d, *J* = 8.8 Hz, 2H), 7.14 (d, *J* = 8.8 Hz, 2H), 6.76 (d, *J* = 8.8 Hz, 2H), 6.71 (d, *J* = 8.8 Hz, 2H), 4.12 (d, *J* = 2.0 Hz, 1H), 3.86 (d, *J* = 2.1 Hz, 1H), 3.78 (s, 3H), 3.73 (s, 3H), 1.74 (s, 3H), 1.66 (s, 3H). ^13^C NMR (DMSO-*d*_6_, 75 MHz) δ: 181.98, 178.34, 159.48, 159.34, 153.07, 135.24, 134.56, 134.39, 133.42, 131.67, 130.45, 125.77, 125.07, 125.07, 122.00, 118.40, 114.68, 114.44, 80.24, 56.09, 55.00, 54.98, 45.93, 27.01. HRMS (ESI^+^) *m*/*z*: calcd for C_29_H_26_NaO_5_S_2_^+^, 541.1119. Found, 541.1134 [M + Na]^+^.

#### 4.1.10 3,4-Bis­((4-fluorophenyl)­thio)-2,2-dimethyl-3,4-dihydro-2*H*-benzo­[*g*]­chromene-5,10-dione (**7f**)

**7f** was obtained as a yellow powder with 37% yield. mp 158.2–158.7 °C. ^1^H NMR (DMSO-*d*_6_, 500 MHz) δ: 8.04–8.02 (m, 1H), 8.02–8.01 (m, 1H), 7.88 (td, *J* = 7.5, 1.5 Hz, 1H), 7.84 (td, *J* = 7.5, 1.5 Hz, 1H), 7.44–7.40 (m, 2H), 7.30–7.27 (m, 2H), 7.09–7.04 (m, 2H), 7.01–6.96 (m, 2H), 4.07 (d, *J* = 1.9 Hz, 1H), 4.04 (d, *J* = 1.9 Hz, 1H), 1.75 (s, 3H), 1.67 (s, 3H). ^13^C NMR (DMSO-*d*_6_, 125 MHz) δ: 182.19, 178.36, 162.09 (d, *J*_C,F_ = 246.8 Hz), 162.06 (d, *J*_C,F_ = 247.2 Hz), 153.28, 135.34 (d, *J*_C,F_ = 70.0 Hz), 135.27 (d, *J*_C,F_ = 58.7 Hz), 134.54, 133.61, 131.68, 130.54, 128.91 (d, *J*_C,F_ = 362.4 Hz), 128.88 (d, *J*_C,F_ = 362.13 Hz), 125.92, 125.74, 117.89, 116.12 (d, *J*_C,F_ = 24.3 Hz), 115.95 (d, *J*_C,F_ = 24.2 Hz), 80.23, 56.16, 46.15, 27.07, 26.84. HRMS (ESI^+^) *m*/*z*: calcd for C_27_H_20_F_2_NaO_3_S_2_^+^, 517.0720. Found, 517.0712 [M + Na]^+^.

#### 4.1.11 2,2-Dimethyl-3,4-bis­((4-(methylthio)­phenyl)­thio)-3,4-dihydro-2*H*-benzo­[*g*]­chromene-5,10-dione (**7g**)

**7g** was obtained as a yellow powder with 73% yield. mp 186.2–186.5 °C. ^1^H NMR (DMSO-*d*_6_, 500 MHz) δ: 8.02–8.01 (m, 1H), 8.01–8.00 (m, 1H), 7.88 (td, *J* = 7.5, 1.5 Hz, 1H), 7.83 (td, *J* = 7.5, 1.5 Hz, 1H), 7.19 (d, *J* = 5.9 Hz, 2H), 7.18 (d, *J* = 6.0 Hz, 2H), 7.11 (d, *J* = 8.5 Hz, 2H), 7.06 (d, *J* = 8.4 Hz, 2H), 4.20 (d, *J* = 1.8 Hz, 1H), 4.05 (d, *J* = 1.8 Hz, 1H), 2.52 (s, 3H), 2.48 (s, 3H), 1.75 (s, 3H), 1.67 (s, 3H). ^13^C NMR (DMSO-*d*_6_, 125 MHz) δ: 181.19, 178.20, 153.04, 138.85, 138.29, 134.32, 133.38, 133.32, 132.42, 131.58, 130.82, 130.42, 127.67, 126.33, 126.16, 125.73, 125.55, 118.05, 80.20, 55.58, 45.35, 26.89, 14.50, 14.41. HRMS (ESI^+^) *m*/*z*: calcd for C_29_H_26_NaO_3_S_4_^+^, 573.0662. Found, 573.0655 [M + Na]^+^.

#### 4.1.12 3,4-Bis­((4-chlorophenyl)­thio)-2,2-dimethyl-3,4-dihydro-2*H*-benzo­[*g*]­chromene-5,10-dione (**7h**)

**7h** was obtained as a yellow powder with 36% yield. mp 175.3–175.8 °C. ^1^H NMR (DMSO-*d*_6_, 500 MHz) δ: 8.03 (t, *J* = 1.8 Hz, 1H), 8.01 (t, *J* = 1.7 Hz, 1H), 7.88 (td, *J* = 7.5, 1.5 Hz, 1H), 7.84 (td, *J* = 7.4, 1.5 Hz, 1H), 7.37 (d, *J* = 8.6 Hz, 2H), 7.30 (d, *J* = 8.6 Hz, 2H), 7.28 (d, *J* = 8.6 Hz, 2H), 7.20 (d, *J* = 8.6 Hz, 2H), 4.14 (d, *J* = 0.8 Hz, 1H), 4.10 (d, *J* = 1.9 Hz, 1H), 1.75 (s, 3H), 1.67 (s, 3H). ^13^C NMR (DMSO-*d*_6_, 125 MHz) δ: 182.12, 178.29, 153.30, 134.49, 134.40, 133.85, 133.75, 133.57, 133.13, 132.98, 131.65, 131.00, 130.51, 128.99, 128.83, 125.88, 125.69, 117.78, 80.29, 55.90, 45.81, 26.89, 26.78. HRMS (ESI^+^) *m*/*z*: calcd for C_27_H_20_Cl_2_NaO_3_S_2_^+^, 549.0129. Found, 549.0129 [M + Na]^+^.

#### 4.1.13 3,4-Bis­((4-hydroxyphenyl)­thio)-2,2-dimethyl-3,4-dihydro-2*H*-benzo­[*g*]­chromene-5,10-dione (**7i**)

**7i** was obtained as a yellow powder with 65% yield. mp 213.3–213.7 °C. ^1^H NMR (DMSO-*d*_6_, 500 MHz) δ: 9.63 (s, 1H), 9.58 (s, 1H), 8.01–8.00 (m, 1H), 7.99–7.98 (m, 1H), 7.87 (td, *J* = 7.5, 1.5 Hz, 1H), 7.82 (td, *J* = 7.5, 1.4 Hz, 1H), 7.09 (d, *J* = 8.6 Hz, 2H), 7.03 (d, *J* = 8.6 Hz, 2H), 6.63 (d, *J* = 8.4 Hz, 4H), 4.20 (d, *J* = 2.6 Hz, 1H), 3.74 (d, *J* = 2.6 Hz, 1H), 1.68 (s, 3H), 1.63 (s, 3H). ^13^C NMR (DMSO-*d*_6_, 125 MHz): δ: 181.84, 178.33, 157.88, 157.60, 152.97, 135.33, 134.51, 134.32, 133.34, 131.66, 130.40, 125.71, 125.56, 122.8, 120.21, 118.99, 116.13, 115.88, 80.53, 55.97, 45.53, 27.18, 26.30. HRMS (ESI^+^) *m*/*z*: calcd for C_27_H_22_NaO_5_S_2_^+^, 513.0807. Found, 513.0806 [M + Na]^+^.

#### 4.1.14 2,2-Dimethyl-3,4-bis­(naphthalen-2-ylthio)-3,4-dihydro-2*H*-benzo­[*g*]­chromene-5,10-dione (**7j**)

**7j** was obtained as a yellow powder with 68% yield. mp 199.2–199.6 °C. ^1^H NMR (DMSO-*d*_6_, 500 MHz) δ: 8.03–8.02 (m, 1H), 8.00–7.98 (m, 1H), 7.84–7.82 (m, 1H), 7.79 (m, 1H), 7.71–7.69 (m, 3H), 7.50–7.36 (m, 7H), 7.32–7.30 (m, 2H), 7,27–7,25 (m, 1H), 4.51 (d, *J* = 2.1 Hz, 1H), 4.27 (d, *J* = 1.7 Hz, 1H), 1.81 (s, 3H), 1.76 (s, 3H). ^13^C NMR (DMSO-*d*_6_, 125 MHz) δ: 181.98, 178.20, 153.16, 134.29, 133.37, 132.83, 132.73, 132.12, 131.76, 131.69, 131.20, 130.43, 129.49, 129.01, 128.36, 128.27, 128.04, 127.17, 126.87, 126.84, 126.30, 126.22, 126.15, 125.98, 125.72, 125.56, 118.18, 80.36, 55.32, 44.85, 26.90. HRMS (ESI^+^) *m*/*z*: calcd for C_35_H_26_NaO_3_S_2_^+^, 581.1221. Found, 581.1234 [M + Na]^+^.

### 4.2 Biological Assay

#### 4.2.1 Cells and Reagents

Human SCC-4, SCC-9, and SCC-25 cells, derived from a human oral tongue SCC (squamous cell carcinoma), were obtained from ATCC (CRL-1624, CRL-1629, and CRL-1628, respectively) and maintained in 1:1 DMEM/F12 (Dulbecco’s modified Eagle’s medium and Ham’s F12 medium; Gibco (Thermo Fisher, Waltham, MA, USA)) supplemented with 10% (v/v) FBS (fetal bovine serum; Invitrogen, Thermo Fisher, Waltham, MA, USA) and 400 ng/mL hydrocortisone (Sigma-Aldrich Corporation, St. Louis, MO, USA). B16-F10 (melanoma; CRL-6475), HCT-116 (colorectal cancer; CCL-247), and HepG2 (liver cancer; HB-8065), in addition to primary normal human gingival fibroblasts obtained from ATCC (PCS-201-018), were maintained in DMEM supplemented with 10% (v/v) of FBS and were used in a maximum of six passages. Cells were grown in a humidified environment containing 5% CO_2_ at 37 °C. For all biological assays, compounds and shikonin were solubilized in 100% DMSO (all Sigma-Aldrich Corp.) to a final concentration of 10 mM and lapachol in ethanol. The carboplatin control was prepared in water (Fauldcarbo; Libbs Farmacêutica, São Paulo, SP, Brazil) and was used as a standard anticancer compound.

#### 4.2.2 Cell Viability Assay (Cytotoxicity)

The viability of OSCC cell lines and primary human fibroblast cells was assessed using the MTT assay as in ref 88. Briefly, cells were grown in triplicate in 96-well plates (5 × 10^3^ cells/well) until confluence. Then, the medium was removed, fresh medium was added, and the cells were returned to the incubator in the presence of different compounds. DMSO at the same concentration was used as a control for 100% cell viability. After 72 h, cells were incubated with 0.5 mg/mL of MTT reagent (3-(4,5-dimethyl-2-thiazolyl)-2,5-diphenyl-2-H-tetrazolium bromide) (Sigma-Aldrich Corporation, Louis, MO, USA) for 3.5 h. Then, the wells containing the cells were washed with PBS heated to 37 °C, the formazan crystals were dissolved in a solvent solution (DMSO/methanol 1:1 v/v), and the absorbance was read at 560 nm using an EPOCH microplate spectrophotometer (BioTek Instruments, Winooski, VT, USA) with the background absorbance at 670 nm subtracted. Control chemotherapeutics (carboplatin and shikonin) were used.

#### 4.2.3 Hemolysis Assay

To determine the surfactant power of the substances present in biological membranes, a hemolysis test was carried out with human blood approved by the Research Ethics Committee of the Universidade Federal Fluminense (CAAE: 43134721.4.0000.5626). Erythrocytes were collected for centrifugation at 1500 rpm for 15 min, washed with PBS (phosphate-buffered saline) supplemented with 10 mM glucose, and counted in an automatic cell counter (Thermo Fisher, Waltham, MA, USA). Erythrocytes were then plated in 96-well plates at a concentration of 4 × 10^8^ cells/well in triplicate, and 10 μL of compounds was added to a final concentration of 300 μM in PBS with glucose (final volume 100 μL). In total, 10 μL of PBS was used as a negative control, and 10 μL of PBS with 0.1% Triton x 100 was used as a positive control. Data reading was performed with EPOCH (BioTek Instruments, Winooski, VT, USA) at an absorbance of 540 nm, and statistical data were generated using GRAPHPAD Prism 8.4.3.0 program (Intuitive Software for Science, San Diego, CA, USA).

#### 4.2.4 *In Vivo* Acute Toxicity Study

The acute toxicity study for compounds **7a** and **7e** was performed in 12 week-old male C57BL/6 mice via intraperitoneal injection and was approved by the University Animal Ethics Board under registration number CEUA no. 2699110419. All experiments were carried out in accordance with Brazilian guidelines and regulations. Dosing and analysis were performed in accordance with 423 OECD guidelines and reviewed by.^35^ Each group of animals had n = 3 and received only one intraperitoneal injection (Day 0) of compounds **7a** and **7e** dissolved in 3 mL of PBS and 3% DMSO. Animals in the control group received only 3% DMSO in PBS. The first dose of the compound was 100 mg/kg. Subsequent dose levels (200 mg/kg and 400 mg/kg) were determined based on the result obtained from the previous dose. Animals were examined daily, twice a day, for mortality and morbidity for 14 days, when all animals were anesthetized (ketamine 100 mg/kg and xylazine 10 mg/kg) followed by cervical dislocation. Macroscopic necropsy of the main organs was performed. The animals’ body weight and average food consumption were measured every 7 days. As an indicator of morbidity, the following signs were evaluated: tremors, seizure, salivation, diarrhea, and lethargy, along with the signs of pain, increased back arching, and mobility disability. The necropsy included examination of the external characteristics of the carcass; external orifices of the body; the abdominal, thoracic, and cranial cavities; and organs/tissues of the liver, thymus, right kidney, right testicle, heart, and lung.

#### 4.2.5 Statistical Analysis and Calculation of IC_50_

Data are presented as means ± SD. IC_50_ values for the MTT assays were obtained by nonlinear regression using the GRAPHPAD 8.4.3.0 program (Intuitive Software for Science, San Diego, CA, USA) from at least three independent experiments. A dose–response curve (inhibitor) vs response using the least-squares method was used to determine the IC_50_, SD, and R2 of the data. The selectivity index was calculated as SI = IC_50_ of the compound in normal oral fibroblast cells/IC_50_ of the same compound for each oral cancer cell line (SCC-4, SCC-9, and SCC-25) and averaged when indicated.

#### 4.2.6 Timelapse Video Microscopy

For cell morphological analysis, 1.5 × 10^5^ cells of the SCC-9 lineage were seeded in a 35 mm Petri dish with supplemented DMEN/F-12 medium and incubated for 24 h in an oven at 37 °C with 5% CO_2_ for grip. Three experimental conditions were carried out: control (DMSO) and treatments with derivatives **7a** and **7e** at a concentration of 2 × IC_50._ After treatment, the plate was transferred to a chamber adapted to a Leica DMi1 inverted optical microscope (Leica Microsystems, Wetzlar, Germany) under controlled CO_2_ and temperature conditions (5% and 37 °C, respectively). For 48 h, phase-contrast images of the same field were captured every minute. Images of each experimental condition were integrated into videos using ImageJ software (National Institute of Health, USA), and different times (indicated) were selected according to the morphological changes observed during the treatment.

#### 4.2.7 Active Caspase

The following reagents were used in this assay: CellEvent Caspase-3/7 Green Ready Probes Reagent kit (R37111, Thermo Fisher, Waltham, Massachusetts, USA). SCC-9 cells were plated in 24-well microplates in the amount of 5 × 10^4^ cells per well as previously described in ref 89. After 24 h in the incubator, the cells were treated with substances **7a** and **7e** at concentrations of 2 x the respective IC_50_, the DMSO control, and the caspase marker reagent (40 μL/500 μL/medium). The duration of the treatment time was 24 h. After this period, the cells were taken to the Zeiss Axio Observer A1 fluorescence optical microscope (Zeiss, Oberkochen, Baden-Württemberg, Germany) to obtain caspase labeling images from five different fields. Data quantification was performed using the ImageJ program, and the values of reactive cells for caspase were compared in relation to the total number of cells per field, and the result was given as a percentage.

#### 4.2.8 Reactive Oxygen Species Production

The assay was performed in SCC-9 cells as previously described.^90^ The wells were then treated with derivatives **7a** and **7e** at a concentration of 2 × IC_50_. Menadione (M9529, Sigma-Aldrich Corporation, San Luis, Mo., USA), at a concentration of 20 μM, served as a positive control, while DMSO, at equivalent concentrations to the test substances, served as the negative control. Cells were then incubated at 37 °C with 5% CO_2_ for the indicated time, and ROS was measured by ROS-Glo H_2_O_2_ Assay G8820 (Promega Corporation, Madison, WI 53711 USA) as indicated by the vendor. The luminescence signal was measured using a luminescence detector, Luminometer TD-20/20 (Turner Designs Instrument, Sunnyvale, CA, USA).

#### 4.2.9 Autophagy Assay

To determine whether compounds **7a** and **7e** induce autophagy, SCC-9 cells were stably transduced with the plasmid expressing LC3-GFP as described.^9^ Briefly, 2.5 × 10^4^ SCC-9-LC3-GFP cells were plated in 500 μL of medium/well and were plated in 24-well plates and 24 h later treated at the IC_50_ concentrations of the two substances and DMSO and incubated. As a positive control, we used compound 8 (Supplementary Figure S1), an already described naphthoquinone-triazole-coumarin hybrid, able to induce autophagy^40^ at its IC_50_ concentration, 30 μM. After 48 h, the cells were visualized using a Zeiss Axio Observer A1 Fluorescence Optical Microscope (Zeiss, Oberkochen, Baden-Württemberg, Germany) to observe the formation of typical autophagy spots and photographed using a high-resolution monochromatic microscopy camera (Axiocam 503 mono, resolution: 1936 × 1460 pixels, pixel size: 4.54 μm × 4.54 μm) to maximize sensitivity and image quality.

#### 4.2.10 Cell Migration Assay

To evaluate the inhibitory capacity of cell migration by substances **7a** and **7e**, 2 × 10^5^ cells of the SCC-9 lineage were plated in a 35 mm Petri dish containing 2.0 mL of supplemented DMEN/F-12 medium and incubated at 37 °C with 5% CO_2_ until they reached at least ∼100% confluence. After this time, the metabolized medium was removed, and the cells were washed once with PBS and fresh medium (2.0 mL) treated with mitomycin C (MMC), 1-amino-9a-metoxi-7-metil-4,7,9,9a-tetra-hidro-3*H*-furo­[3,4:6,7]­nafto­[1,2-*d*]­imidazole-2­(1*H*)-ona (Sigma-Aldrich Corporation, San Luis, Missouri, USA) at a concentration of 0.5 mg/mL, in a volume of 0.5 μL/mL, and returned to the incubator for 2 h. At the end of the time, scratch wounds were created on the cell monolayer by using a 200 μL capacity tip. After washing the cells 3 times with PBS, treatment with substances **7a** and **7e** and the DMSO control was performed at a sublethal concentration of 1/8 of the IC_50_ (3.3 and 5.8 μM, respectively). The plate containing the cells was inserted into a chamber adapted to a Leica DMi1 inverted optical microscope (Leica Microsystems, Wetzlar, Germany) under controlled conditions of CO_2_ (5%) and temperature (37 °C). Phase contrast images in timelapse footage at 20× magnification for 72 h were captured every 2 min and 24 s. Measurements between the captured margins were collected using ImageJ analysis software (National Institute of Health, USA). An average of 5 different distances between the edges was obtained for each time. At the initial time of each situation, the distance between the edges of the wound was defined as 100%, representing the open wound. The percentage of wound closure at each subsequent time is calculated in relation to the initial distance (time: 0 h).

### 4.3 *In Silico* Studies

#### 4.3.1 Drawing of the Molecular Structures of Substances **7a** and **7e**

For the *in silico* evaluation, the molecular structures of derivatives **7a** and **7e** were drawn using the Avogadro chemical structure drawing tool available on the web (https://avogadro.cc/) to obtain their three-dimensional (3D) structure. After this step, the SMILES (Simplified Molecular Input Line Entry Specification) of the two molecules was generated in a PDB (Protein Data Bank) format file. The OpenBabelGUI software was used to convert the PDB file into the SMILES extension file, which was subsequently saved on the computer for application in computational pharmacokinetic evaluations. The SMILES structures of carboplatin and doxorubicin were obtained from the web (https://pubchem.ncbi.nlm.nih.gov/).

#### 4.3.2 Prediction of Physicochemical Properties, Pharmacokinetics, and Toxicity

To predict the similarity of molecules **7a** and **7e** and the pharmacokinetic behavior based on their chemical structures and seek information on ADME (absorption, distribution, metabolism, and excretion) classification and toxicity (T) predictions, the following free pharmacokinetic analysis servers found on the web were used: admetSAR (http://lmmd.ecust.edu.cn/admetsar1), ADMETlab 2.0 (https://admetmesh.scbdd.com/), OSIRIS (https://www.organic-chemistry.org/prog/peo/), pkCSM (https://biosig.lab.uq.edu.au/pkcsm/), PROTOX 3.0 (https://comptox.charite.de/protox3/), and SwissADME (http://www.swissadme.ch/). To evaluate the physicochemical parameters of the two molecules, we used the Lipinski Rule of Five (RO5) criteria, which uses specific parameters and provides an output in the form of a violation score for each criterion. We use the server SwissADME when searching for ratings for Lipinski’s Rule of Five. The structure in SMILES format saved in a SMILES extension file was used as input, and the output is a series of physicochemical parameters bringing classifications known as ADME/T. In addition to the characterization of the new molecules, carboplatin and doxorubicin were also subjected to the same tests for comparison purposes.

#### 4.3.3 Molecular Modeling Tools for Target Prediction

Compound **7e** was subjected to molecular docking studies to predict its molecular target. Since compound **7e** was tested in an experimental assay as a mixture of antistereoisomers, all of them were considered for the docking studies. They were drawn and optimized using the Spartan’10 software (Wave function Inc., Irvine, CA, USA). Then, a conformational analysis was performed using the MMFF force field, and the conformer with the lowest energy was optimized using the semiempirical PM3 method. Furthermore, an energy calculation was carried out using the Hartree–Fock method with the 6-31G* basis set (HF/6-31G*). The optimized structures and the electrostatic charges were used as input for docking studies. Anticancer compounds with known mechanisms of action, such as shikonin, lapachol, and the drug doxorubicin (DOX), were used for comparative purposes.

The three-dimensional structures of putative targets were obtained from the Protein Data Bank (PDB) under the following codes: ATPase domain of topoisomerase IIα (PDB 1ZXM), ribosomal protein S6 kinase 2 (RSK2; PDB 4NW6), topoisomerase I DNA-binding domain (PDB 1K4T), human pyruvate kinase M2 (PKM2; PDB 3SRD), topoisomerase IIα (PDB 5GWK), and topoisomerase IIβ (PDB 3QX3).

Molecular docking studies were performed with Autodock Tools 1.5.7 and Autodock Vina 1.1.2 software.^91^ The proteins were prepared by removing solvents and artifacts and adding polar hydrogens and Gasteiger charges, and they were maintained as rigid structures. The ligands were maintained in a flexible state, with their torsional bonds automatically defined using Autodock Tools version 1.5.7. The docking procedures used were previously validated by our group.^8,9,92^

The poses with the lowest binding energy were visually inspected, and their interactions were analyzed using Discovery Studio Visualizer 2021 ^93^ and PyMOL v. 2.0 (The PyMOL Molecular Graphics System, Version 2.0 Schrodinger, LLC).

### 4.4 Use of IA

During the preparation of this work, the authors used ChatGPT version 4.0 to improve the English language of parts of the article. Upon generating draft language, the authors reviewed, edited, and revised the language to their own liking and take ultimate responsibility for the content of this publication. No information or research included in this work was retrieved from the IA databases.

## Supplementary Material







## Data Availability

Data will be available upon request.

## Notes

Video S1: Cell death morphology time-lapse experiment. Time-lapse imaging of SCC-9 cells treated with derivatives **7a** and **7e** (2 × IC_50_) for 48 hours (as described in methods) revealed pronounced morphological changes consistent with apoptotic cell death. Treated cells exhibited cell shrinkage, membrane blebbing, and progressive detachment from the plate surface, while control cells (DMSO) maintained stable morphology and high level of proliferation throughout the experiment. These observations suggest that both compounds effectively trigger cell death pathways with distinct apoptotic phenotypes over time (MP4)
Video S2: Cell migration (scratch assay) time-lapse experiment. Time-lapse imaging of SCC-9 cell migration during a “scratch wound” assay over 72 hours demonstrated that treatment with derivatives **7a** and **7e** (1/8 × IC_50_) (as described in methods) modulated the migratory behavior of the cells to different extents. Compared with the DMSO control, both compounds delayed wound closure, indicating a potential inhibitory effect on cellular motility. Quantitative analysis of wound closure confirmed varying degrees of migration inhibition, highlighting the differential impact of these compounds on processes related to cell movement and potentially metastatic behavior (MP4)
Structure of compound **8** and spectral data (^1^H and ^13^C NMR and HRMS spectra) of **2** and **7a–j** (PDF)
