# Supplementary material for: Mechanistic Insights into the Anticancer Action of Novel 2‑Hydroxy-1,4-naphthoquinone Thiol Derivatives
Source: ACS Omega. 2025 Sep 5;10(36):41975–92. doi: 10.1021/acsomega.5c06730 (PMC12444508; doi:10.1021/acsomega.5c06730)
Supplement: Supplementary file 3 [file ao5c06730_si_003.pdf]

Mechanistic Insights into the Anticancer Action of Novel 2-Hydroxy-1,4-Naphthoquinone Thiol Derivatives

Thaís Barreto Santos[1†], Alex de Souza Cruz Lopes Canuto[2†], João Francisco Blaudt Virgilio de Carvalho Meira[3], Ana Caroline Santos-Diniz[4], Rafaella Machado de Assis Cabral Ribeiro[1], Caroline Reis Santiago Paschoal[5,6], Vitor Won-Held Rabelo[6], Paula Alvarez Abreu[6], Vitor Francisco Ferreira[,7], David Rodrigues da Rocha[1*], Bruno Kaufmann Robbs[4*]

[1] Department of Chemistry, Universidade Federal Fluminense, Institute of Chemistry, 24020-141 Niterói-RJ, Brazil

[2] Postgraduate Program in Sciences Applied to Health Products, Universidade Federal Fluminense, Faculty of Pharmacy, 24241-000 Niterói-RJ, Brazil

[3] Postgraduate Program in Morphological Sciences, Universidade Federal do Rio de Janeiro, Institute of Biomedical Sciences, 21941-590 Rio de Janeiro-RJ, Brazil.

[4] Department of Basic Sciences, Nova Friburgo Institute of Health (ISNF), Universidade Federal Fluminense, 28625-650 Nova Friburgo-RJ, Brazil

[5] Postgraduate Program in Pharmaceutical Sciences, Universidade Federal do Rio de Janeiro, Institute of Biodiversity and Sustainability, 27965-045 Macaé-RJ, Brazil.

[6] Institute of Biodiversity and Sustainability, Universidade Federal do Rio de Janeiro, 27965-045 Macaé-RJ, Brazil.

[7] Department of Pharmaceutical Technology, Universidade Federal Fluminense, Faculty of Pharmacy, 24241-000 Niterói-RJ, Brazil

Corresponding Authors

David Rodrigues da Rocha – Department of Chemistry, Universidade Federal Fluminense, Institute of Chemistry, 24020-141 Niterói-RJ, Brazil; Email: davidrrocha@id.uff.br

Bruno Kaufmann Robbs – Department of Basic Sciences, Nova Friburgo Institute of Health (ISNF), Universidade Federal Fluminense, 28625-650 Nova Friburgo-RJ, Brazil; Email: brunokr@id.uff.br

[†] *These authors contributed equally to this work*

SUPPORTING INFORMATION

Contents	Page
Figure S1. Structure of compound **8**	S1
HRMS of the final compounds **7a-j**	S2-11
^{1}H-NMR spectra of intermediate compound **2**	S12
^{1}H- and ^{13}C-NMR spectra of the final compounds **7a-j**	S13-23

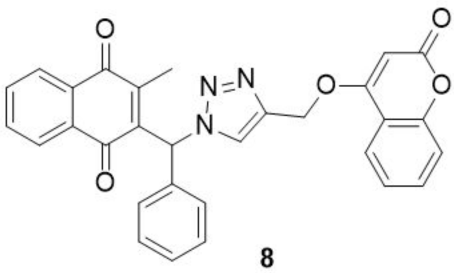

8

Figure S1. Coumarin-naphthoquinone **8**

Acquisition Parameter

Source Type	ESI	Ion Polarity	Positive	Set Nebulizer	0.4 Bar
Focus	Not active	Set Capillary	4000 V	Set Dry Heater	200 °C
Scan Begin	100 m/z	Set End Plate Offset	-400 V	Set Dry Gas	4.0 l/min
Scan End	1000 m/z	Set Charging Voltage	2000 V	Set Divert Valve	Source
		Set Corona	0 nA	Set APCI Heater	0 °C

+MS, 0.4-0.6min #22-37

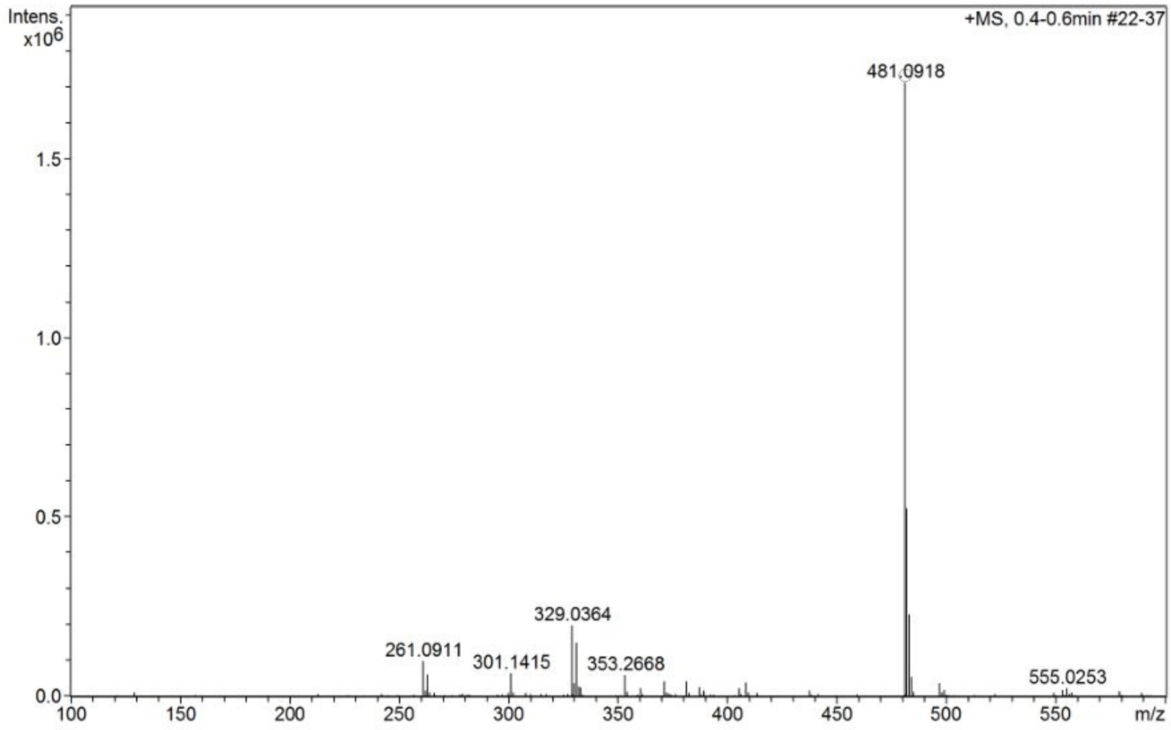

Figure S2. HRMS of **7a**

Acquisition Parameter

Source Type	ESI	Ion Polarity	Positive	Set Nebulizer	0.4 Bar
Focus	Not active	Set Capillary	4000 V	Set Dry Heater	200 °C
Scan Begin	100 m/z	Set End Plate Offset	-400 V	Set Dry Gas	4.0 l/min
Scan End	1000 m/z	Set Charging Voltage	2000 V	Set Divert Valve	Source
		Set Corona	0 nA	Set APCI Heater	0 °C

+MS, 0.4-0.6min #25-33

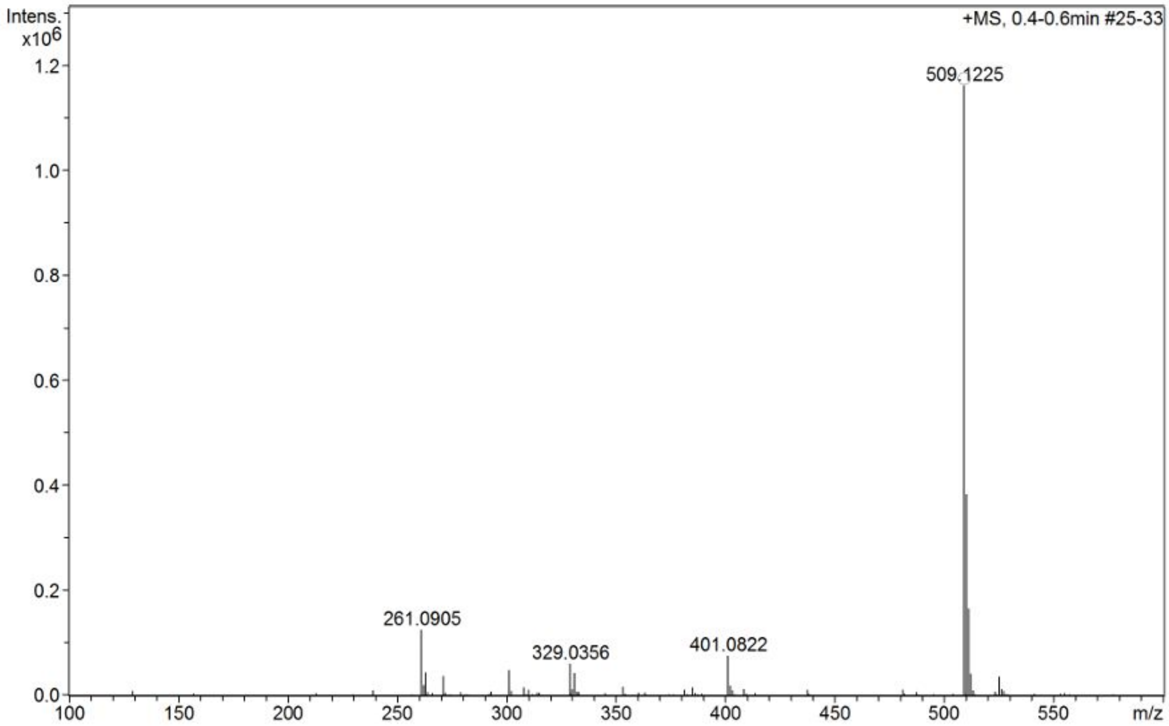

Figure S3. HRMS of **7b**

Acquisition Parameter

Source Type	ESI	Ion Polarity	Positive	Set Nebulizer	0.4 Bar
Focus	Not active	Set Capillary	4000 V	Set Dry Heater	200 °C
Scan Begin	100 m/z	Set End Plate Offset	-400 V	Set Dry Gas	4.0 l/min
Scan End	1000 m/z	Set Charging Voltage	2000 V	Set Divert Valve	Source
		Set Corona	0 nA	Set APCI Heater	0 °C

+MS, 0.4-0.7min #24-41

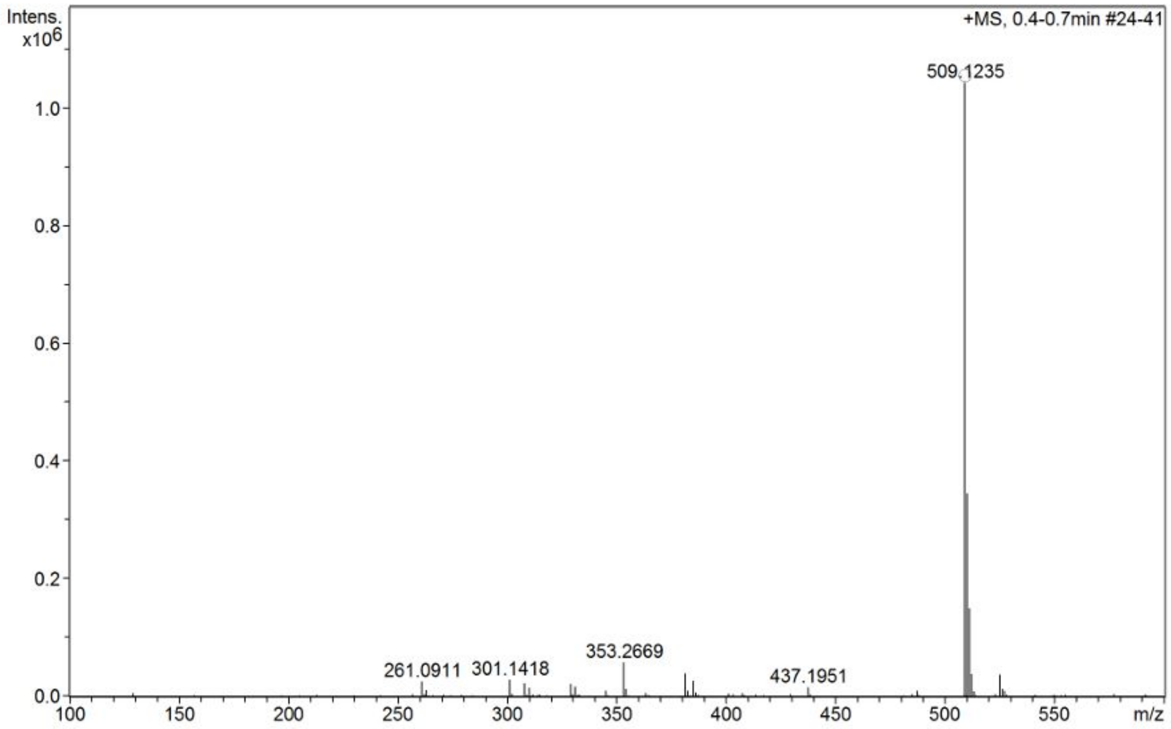

Figure S4. HRMS of **7c**

Acquisition Parameter

Source Type	ESI	Ion Polarity	Positive	Set Nebulizer	0.4 Bar
Focus	Not active	Set Capillary	4000 V	Set Dry Heater	200 °C
Scan Begin	100 m/z	Set End Plate Offset	-400 V	Set Dry Gas	4.0 l/min
Scan End	1000 m/z	Set Charging Voltage	2000 V	Set Divert Valve	Source
		Set Corona	0 nA	Set APCI Heater	0 °C

+MS, 0.5-0.7min #29-41

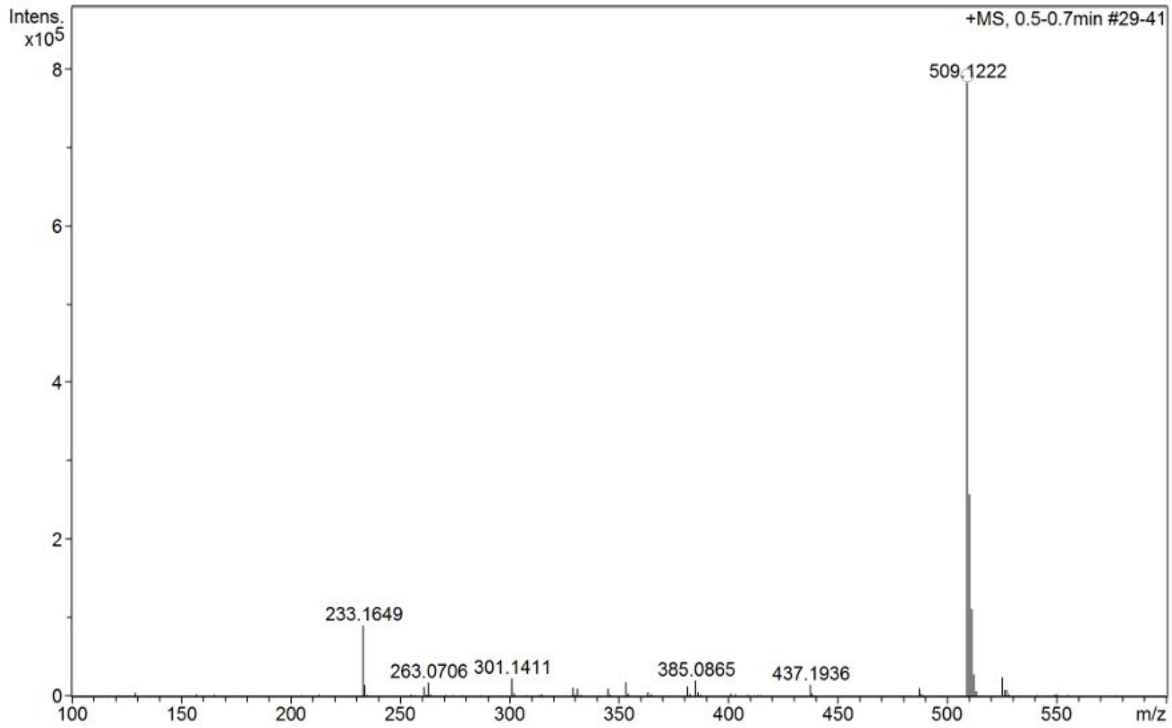

Figure S5. HRMS of **7d**

Acquisition Parameter

Source Type	ESI	Ion Polarity	Positive	Set Nebulizer	0.4 Bar
Focus	Not active	Set Capillary	4000 V	Set Dry Heater	200 °C
Scan Begin	100 m/z	Set End Plate Offset	-400 V	Set Dry Gas	4.0 l/min
Scan End	1000 m/z	Set Charging Voltage	2000 V	Set Divert Valve	Source
		Set Corona	0 nA	Set APCI Heater	0 °C

+MS, 0.4-0.7min #22-38

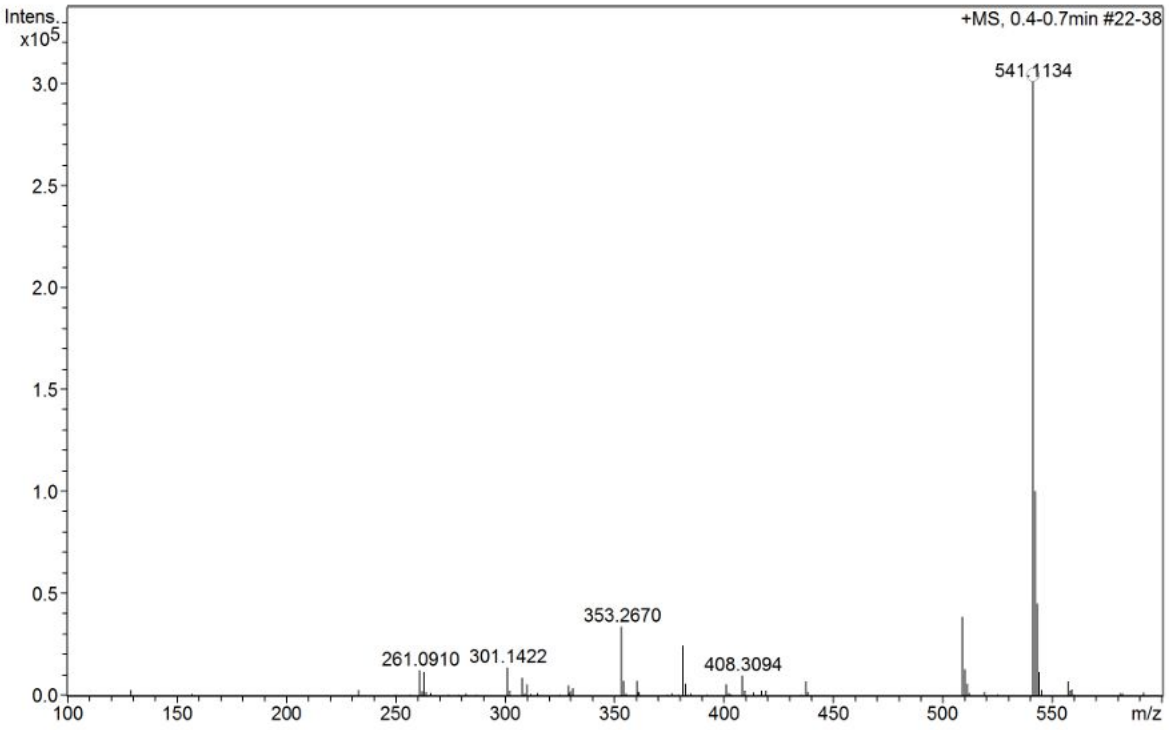

Figure S6. HRMS of **7e**

Acquisition Parameter

Source Type	ESI	Ion Polarity	Positive	Set Nebulizer	0.4 Bar
Focus	Not active	Set Capillary	4000 V	Set Dry Heater	200 °C
Scan Begin	100 m/z	Set End Plate Offset	-400 V	Set Dry Gas	4.0 l/min
Scan End	1000 m/z	Set Charging Voltage	2000 V	Set Divert Valve	Source
		Set Corona	0 nA	Set APCI Heater	0 °C

+MS, 0.5-0.8min #30-45

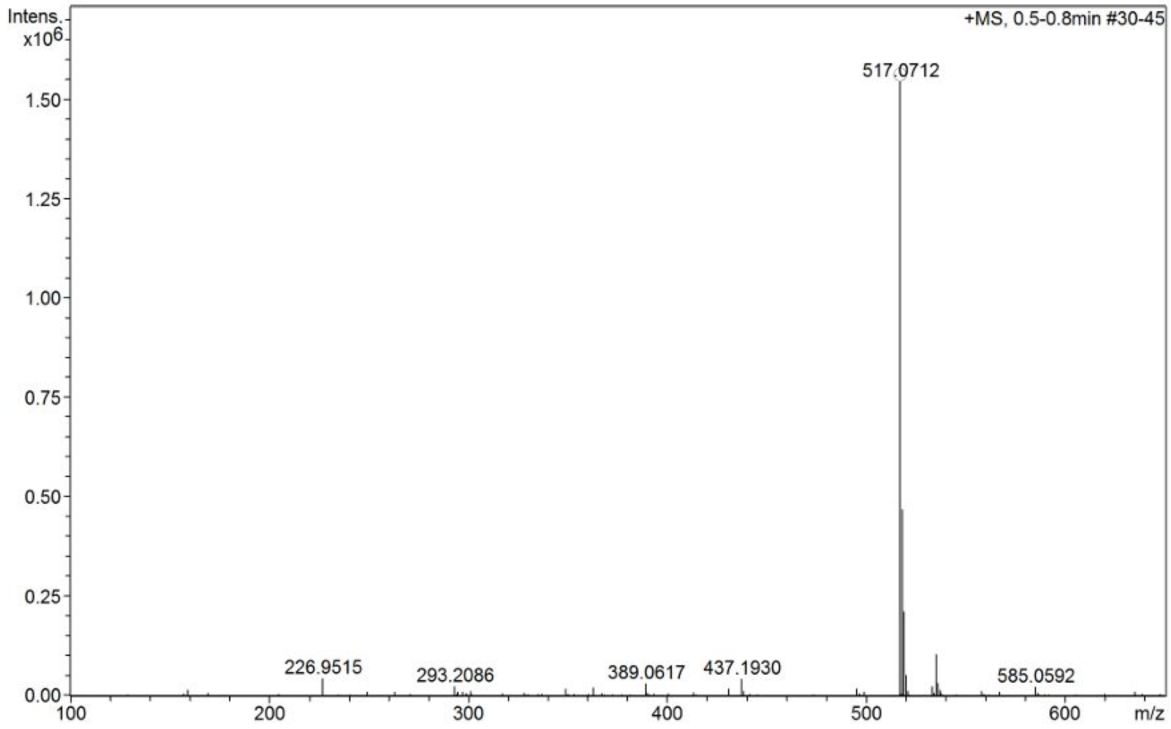

Figure S7. HRMS of **7f**

Acquisition Parameter					
Source Type	ESI	Ion Polarity	Positive	Set Nebulizer	0.4 Bar
Focus	Not active	Set Capillary	4000 V	Set Dry Heater	200 °C
Scan Begin	100 m/z	Set End Plate Offset	-400 V	Set Dry Gas	4.0 l/min
Scan End	1000 m/z	Set Charging Voltage	2000 V	Set Divert Valve	Source
		Set Corona	0 nA	Set APCI Heater	0 °C

+MS, 0.3-0.6min #15-32

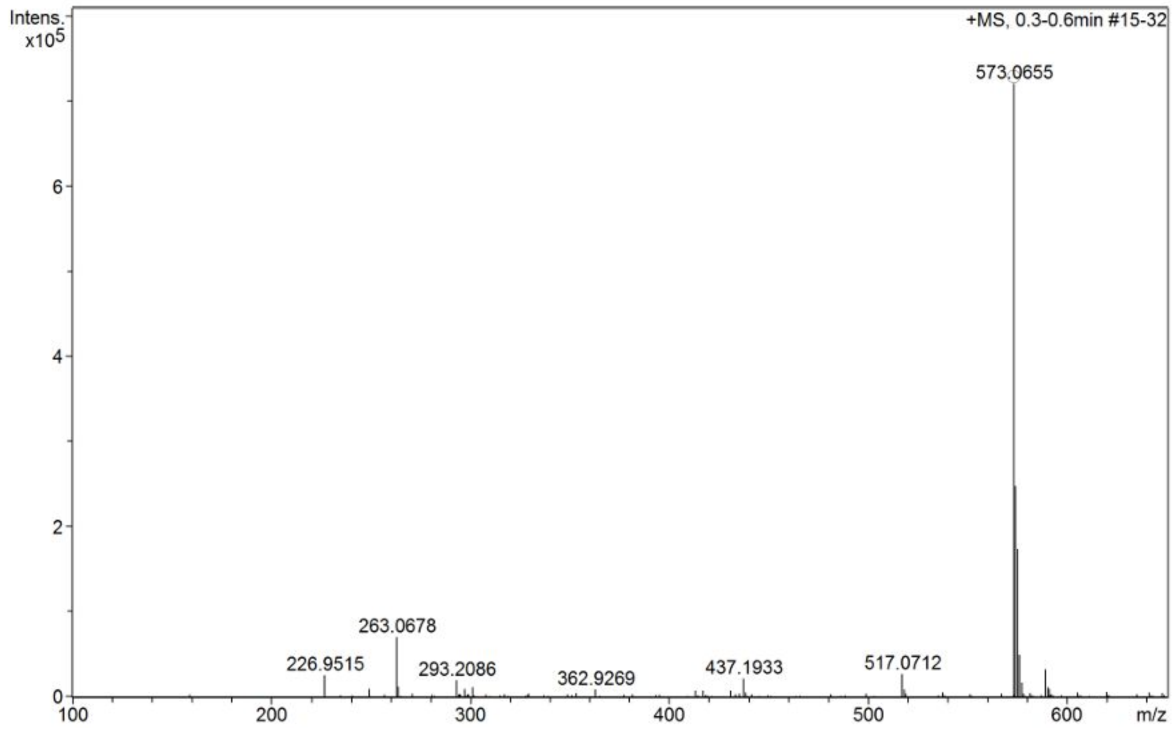

Figure S8. HRMS of **7g**

Acquisition Parameter					
Source Type	ESI	Ion Polarity	Positive	Set Nebulizer	0.4 Bar
Focus	Not active	Set Capillary	4000 V	Set Dry Heater	200 °C
Scan Begin	100 m/z	Set End Plate Offset	-400 V	Set Dry Gas	4.0 l/min
Scan End	1000 m/z	Set Charging Voltage	2000 V	Set Divert Valve	Source
		Set Corona	0 nA	Set APCI Heater	0 °C

+MS, 0.3-0.5min #16-30

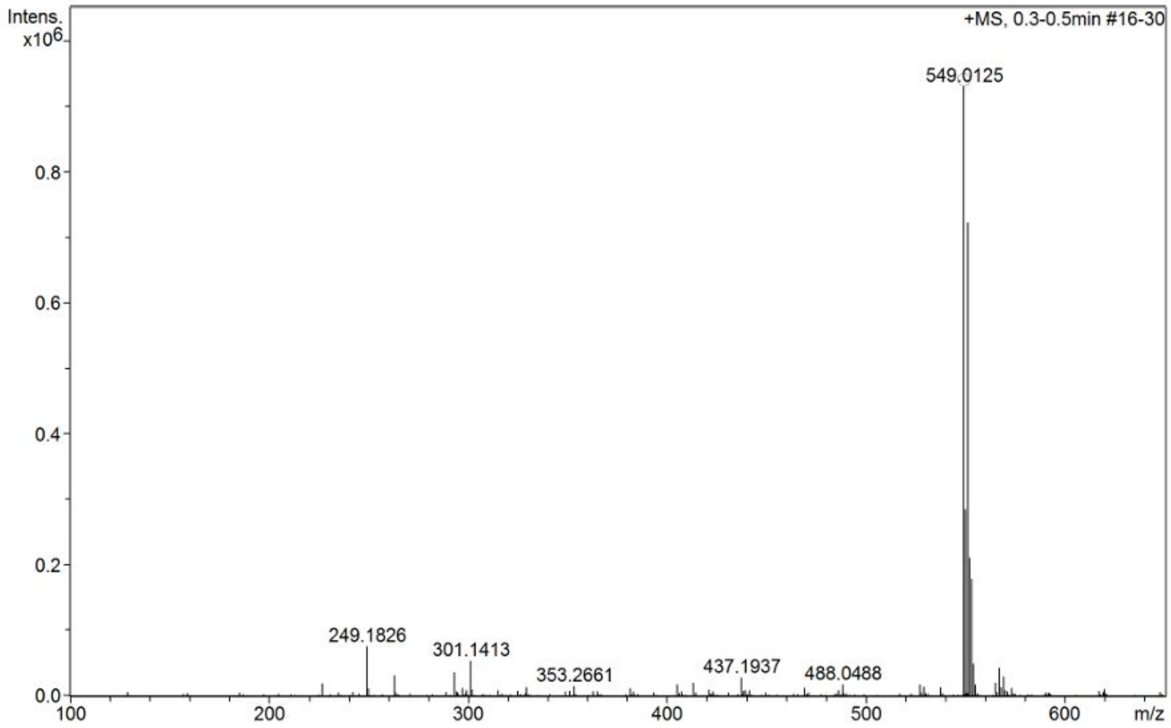

Figure S9. HRMS of **7h**

Acquisition Parameter

Source Type	ESI	Ion Polarity	Positive	Set Nebulizer	0.4 Bar
Focus	Not active	Set Capillary	4000 V	Set Dry Heater	200 °C
Scan Begin	100 m/z	Set End Plate Offset	-400 V	Set Dry Gas	4.0 l/min
Scan End	1000 m/z	Set Charging Voltage	2000 V	Set Divert Valve	Source
		Set Corona	0 nA	Set APCI Heater	0 °C

+MS, 0.5-0.8min #31-49

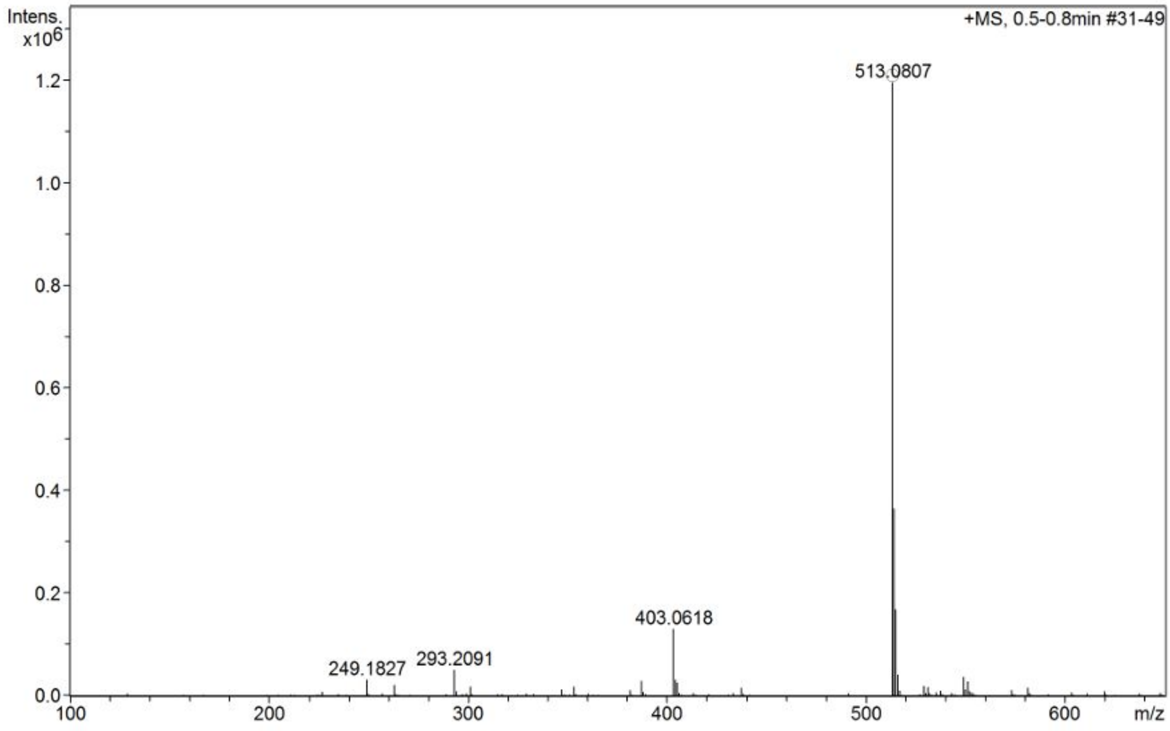

Figure S10. HRMS of **7i**

Acquisition Parameter

Source Type	ESI	Ion Polarity	Positive	Set Nebulizer	0.4 Bar
Focus	Not active	Set Capillary	4000 V	Set Dry Heater	200 °C
Scan Begin	100 m/z	Set End Plate Offset	-400 V	Set Dry Gas	4.0 l/min
Scan End	1000 m/z	Set Charging Voltage	2000 V	Set Divert Valve	Source
		Set Corona	0 nA	Set APCI Heater	0 °C

+MS, 0.3-0.7min #20-38

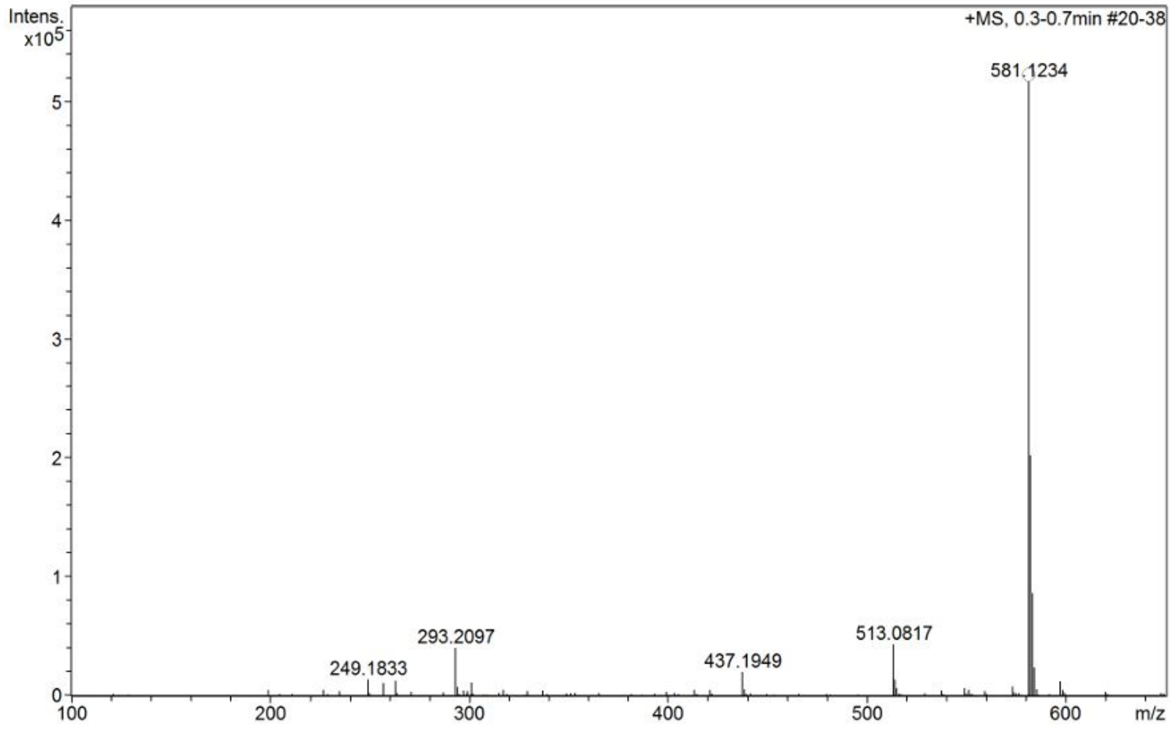

Figure S11. HRMS of **7j**

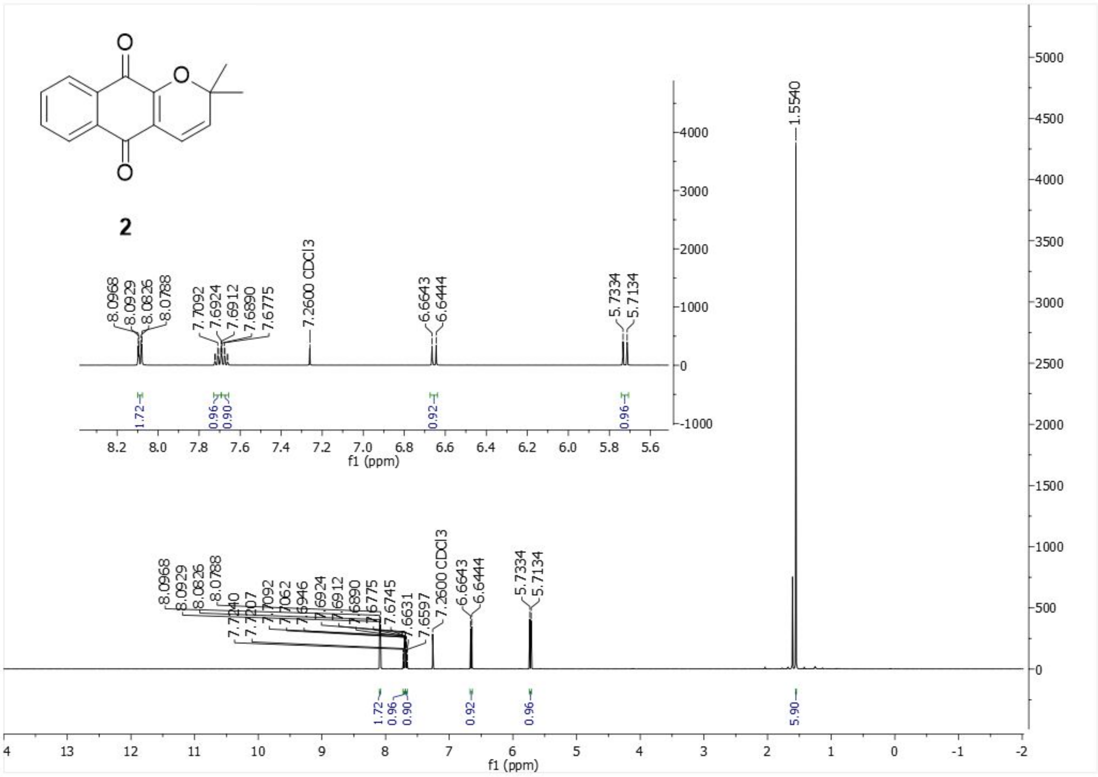

Figure S12.^{1}H NMR (500 MHz, $CDCl_3$) of **2**

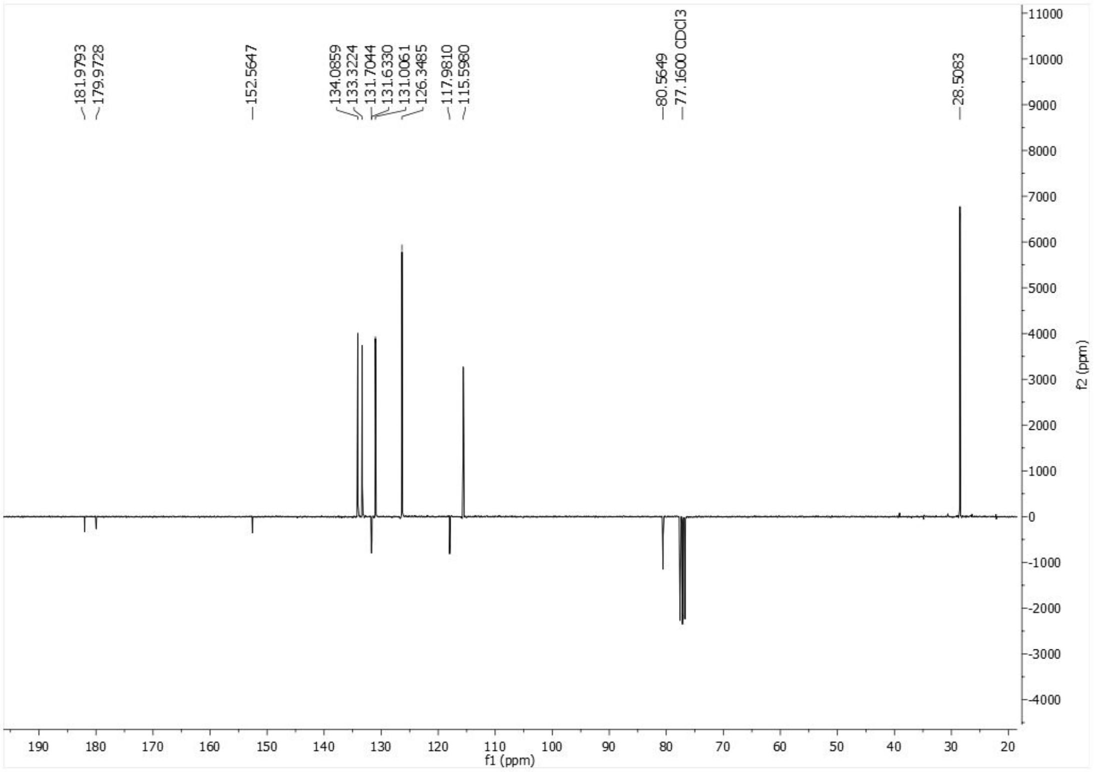

Figure S13.^{13}C/APT NMR (125 MHz, $CDCl_3$) of **2**

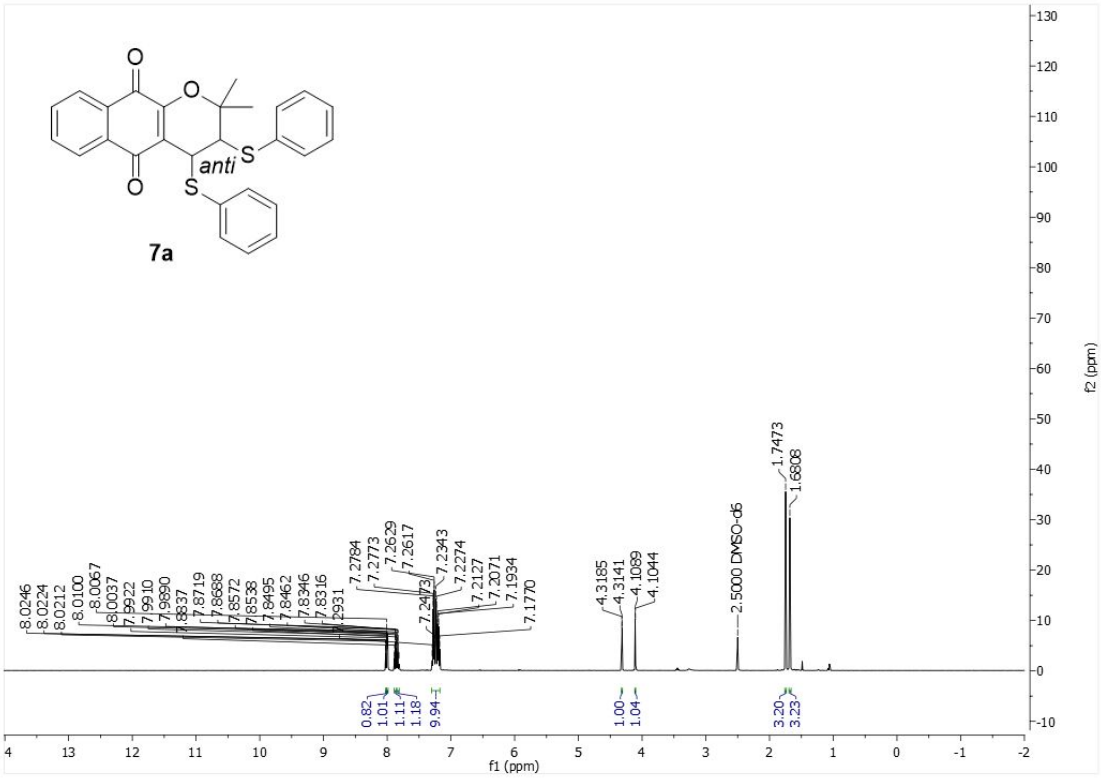

Figure S14.^{1}H NMR (500 MHz, DMSO-d_6) of **7a**

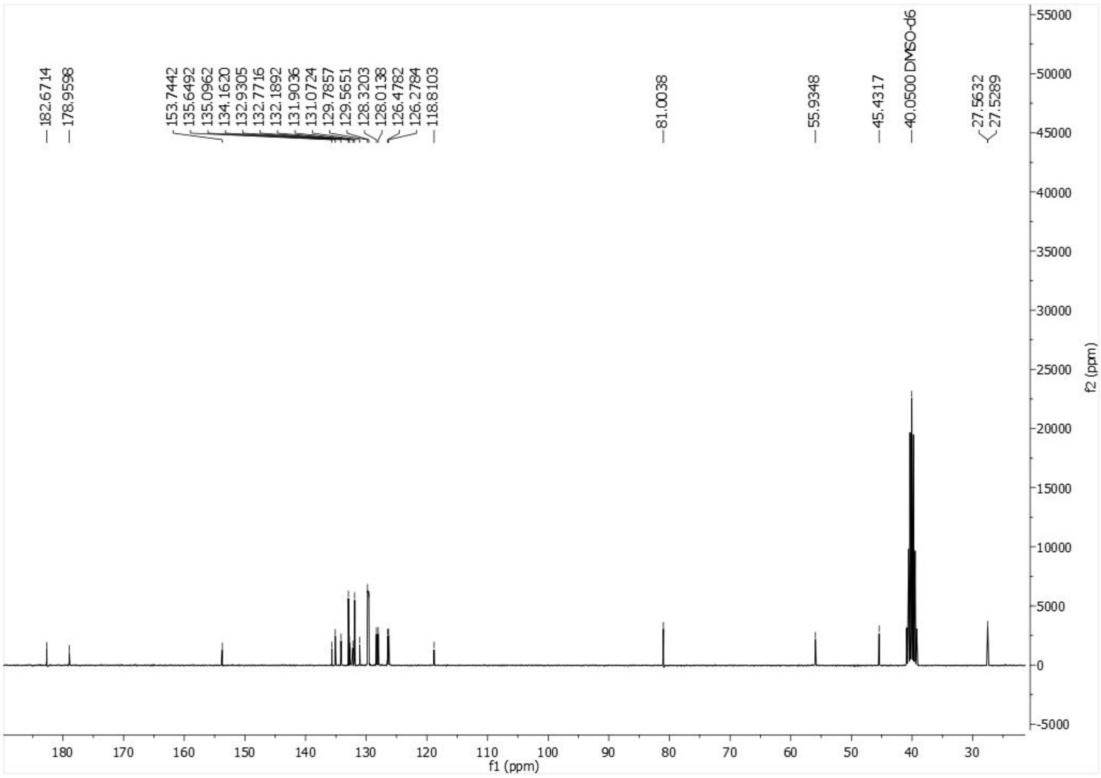

Figure S15.^{13}C NMR (75 MHz, DMSO-d_6) of **7a**

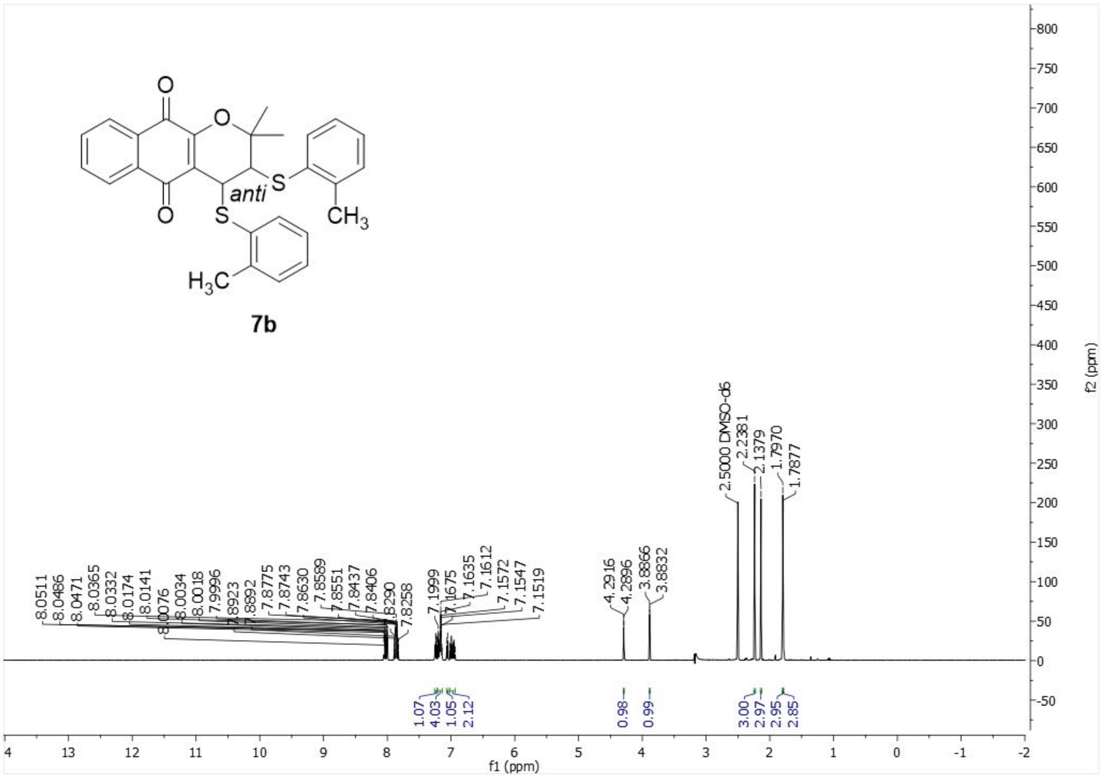

Figure S16.^{1}H NMR (500 MHz, DMSO-d_6) of **7b**

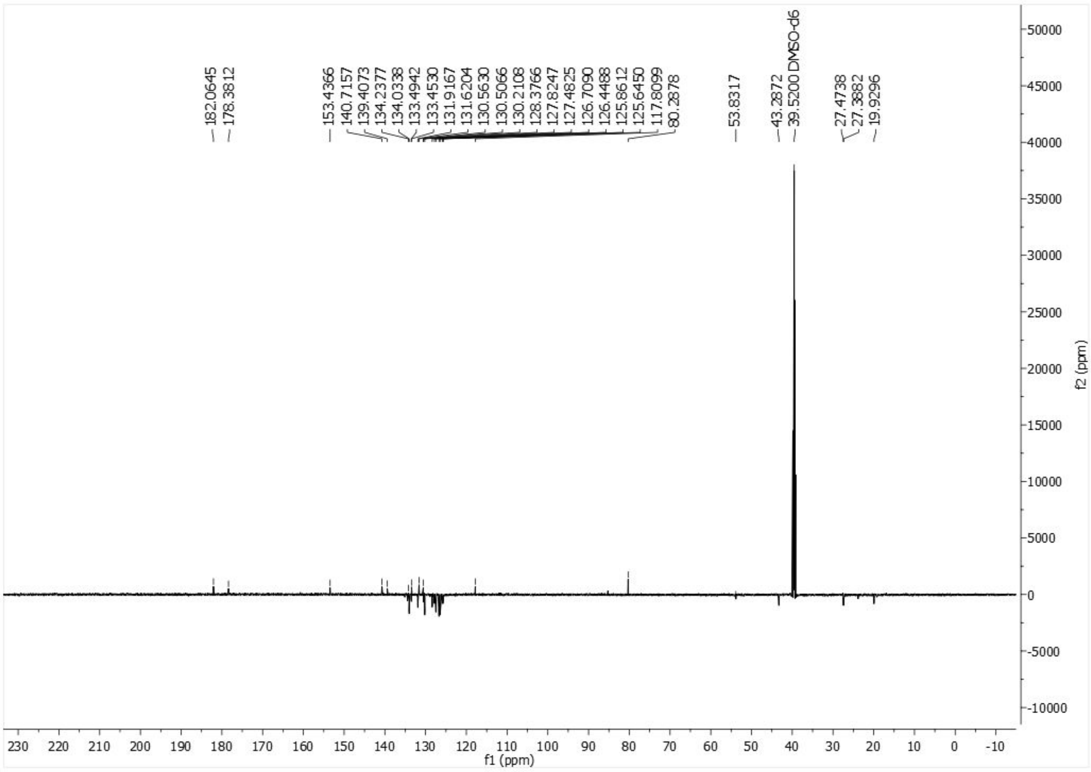

Figure S17.^{13}C/APT NMR (125 MHz, DMSO-d_6) of **7b**

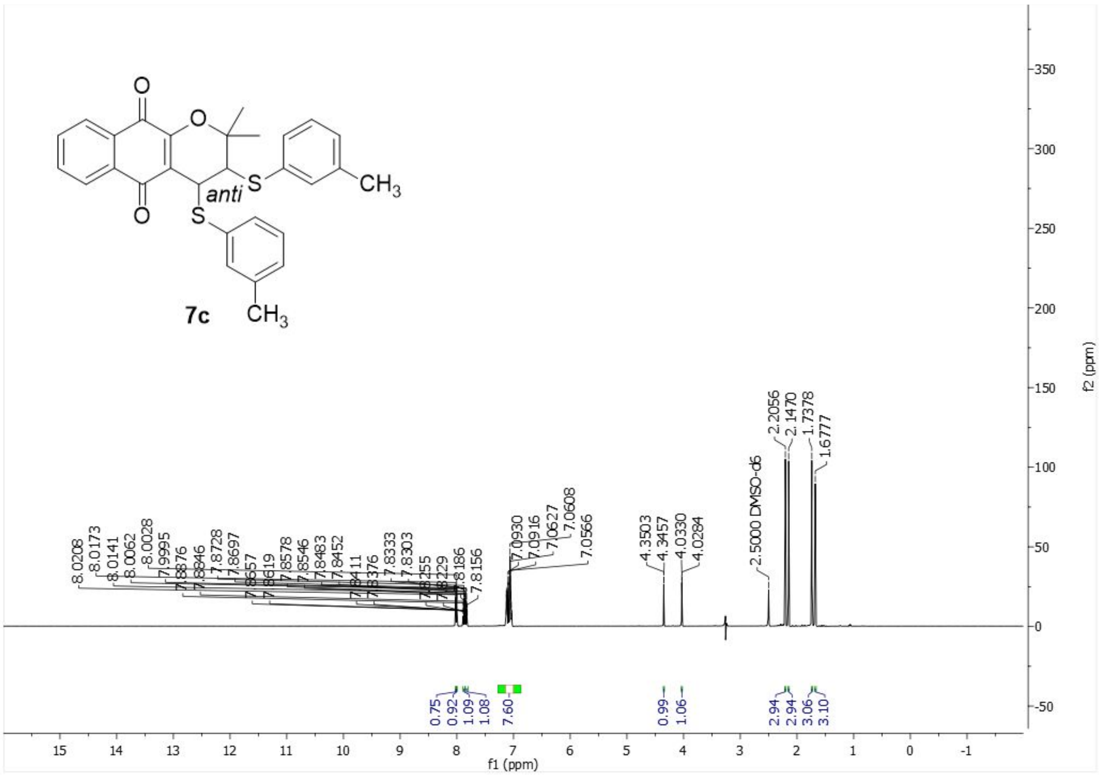

Figure S18.^{1}H NMR (500 MHz, $CDCl_3$) of **7c**

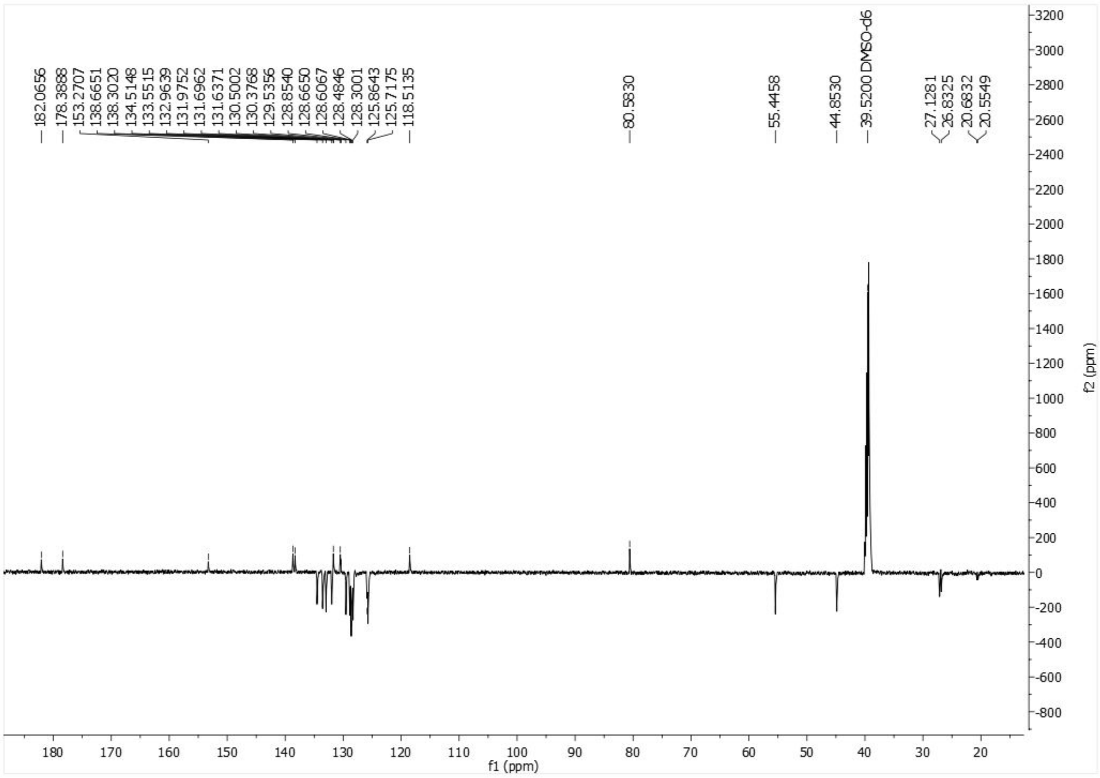

Figure S19.^{13}C/APT NMR (125 MHz, $CDCl_3$) of **7c**

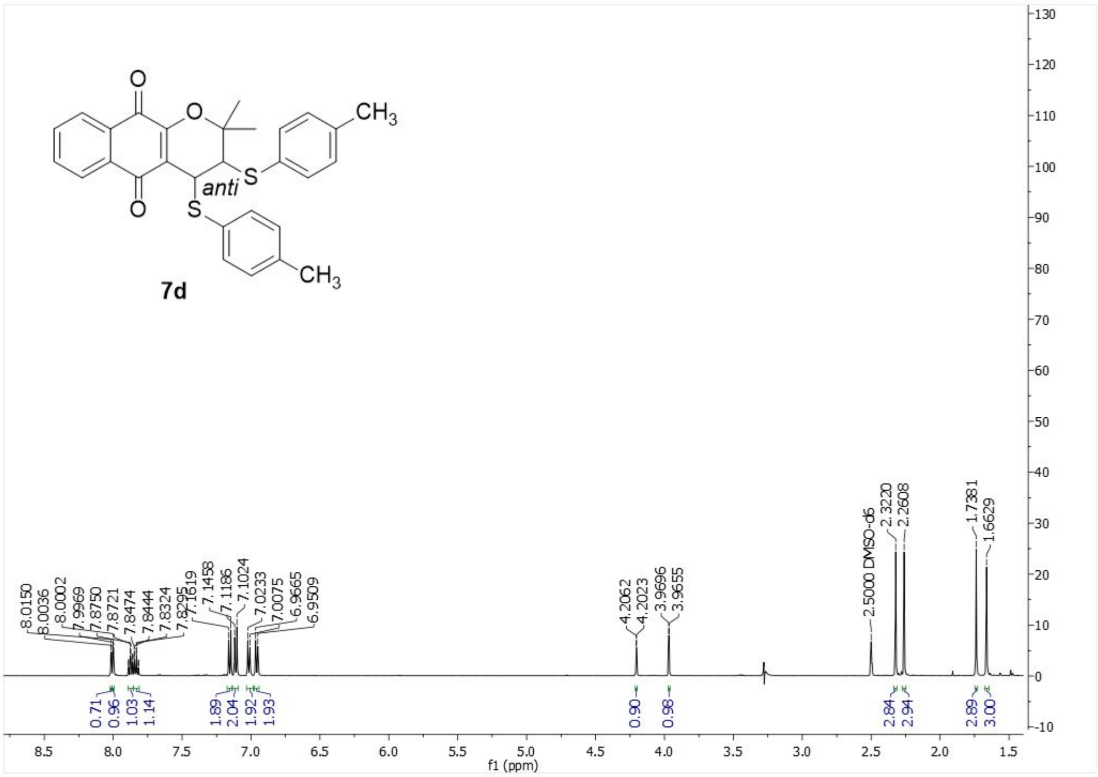

Figure S20.^{1}H NMR (500 MHz, DMSO-d_6) of **7d**

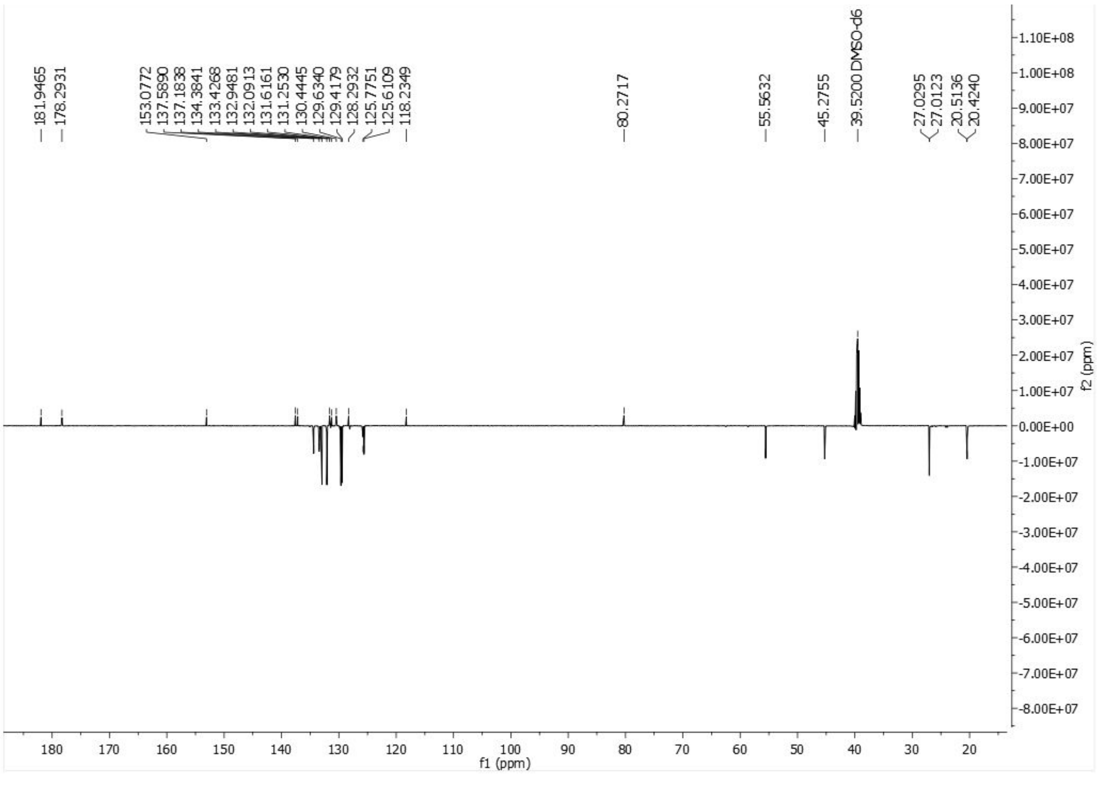

Figure S21.^{13}C/APT NMR (125 MHz, DMSO-d_6) of **7d**

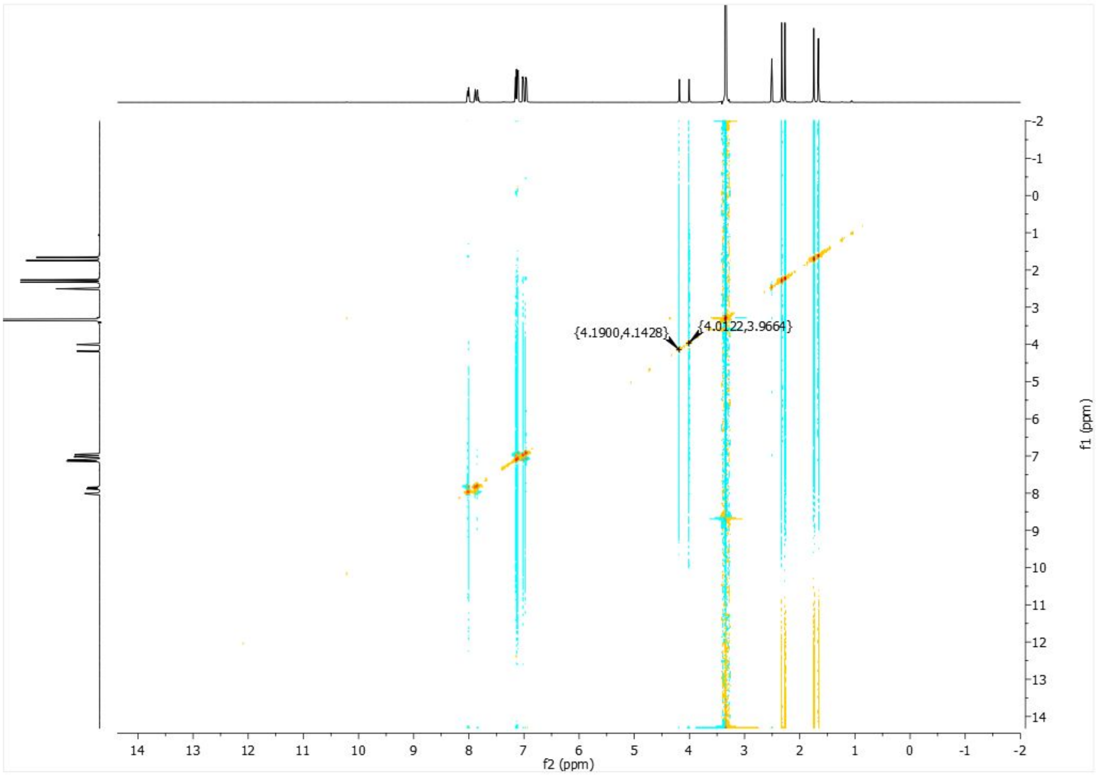

Figure S22. (^{1}H-^{1}H)-NOESY NMR (500 MHz, DMSO-d_6) of **7d**

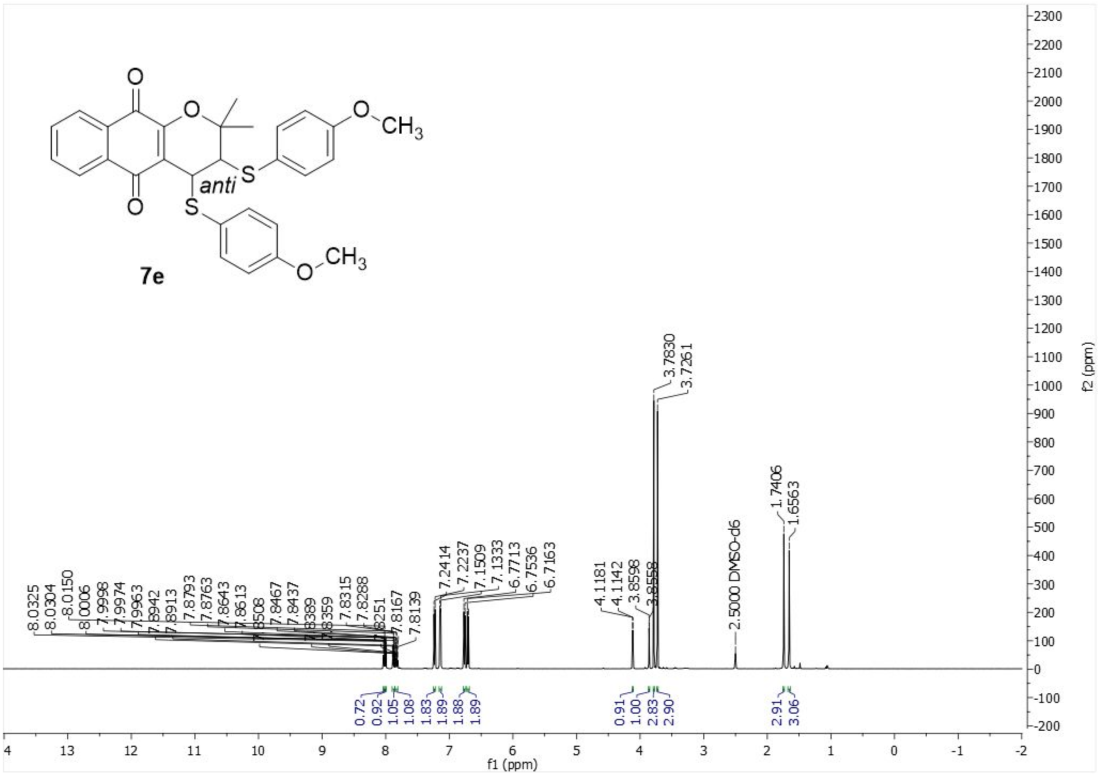

Figure S23.^{1}H NMR (500 MHz, DMSO-d_6) of **7e**

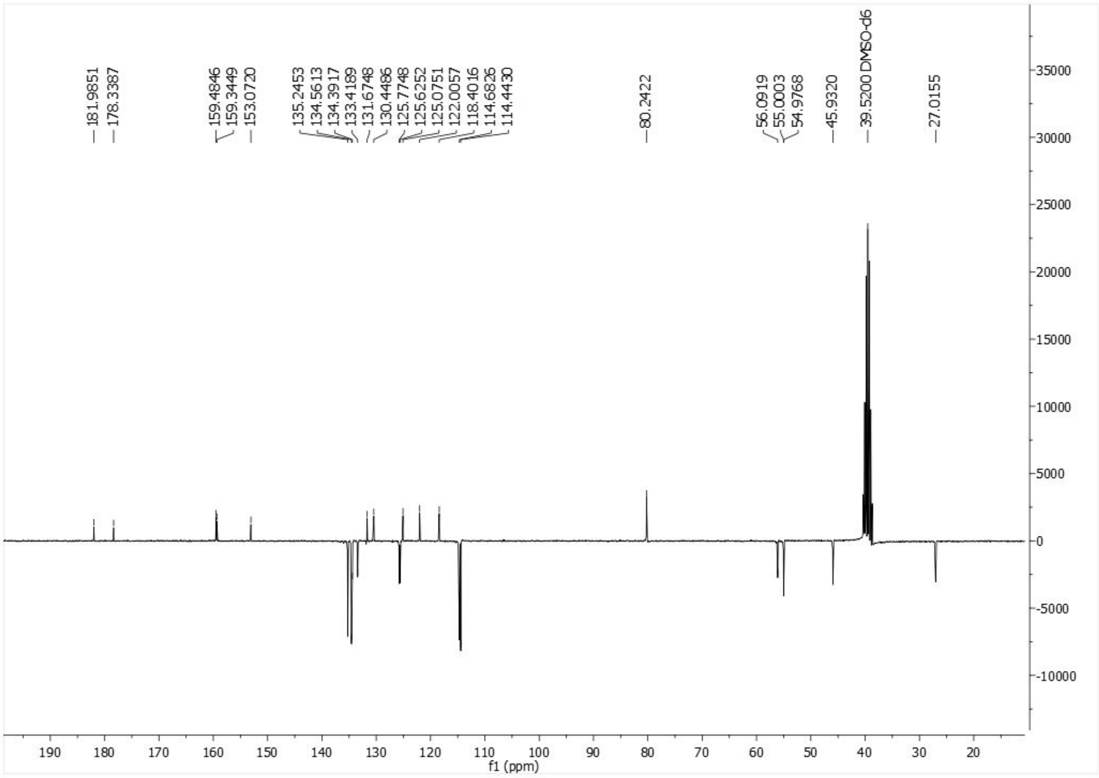

Figure S24.^{13}C/APT NMR (75 MHz, DMSO-d_6) of **7e**

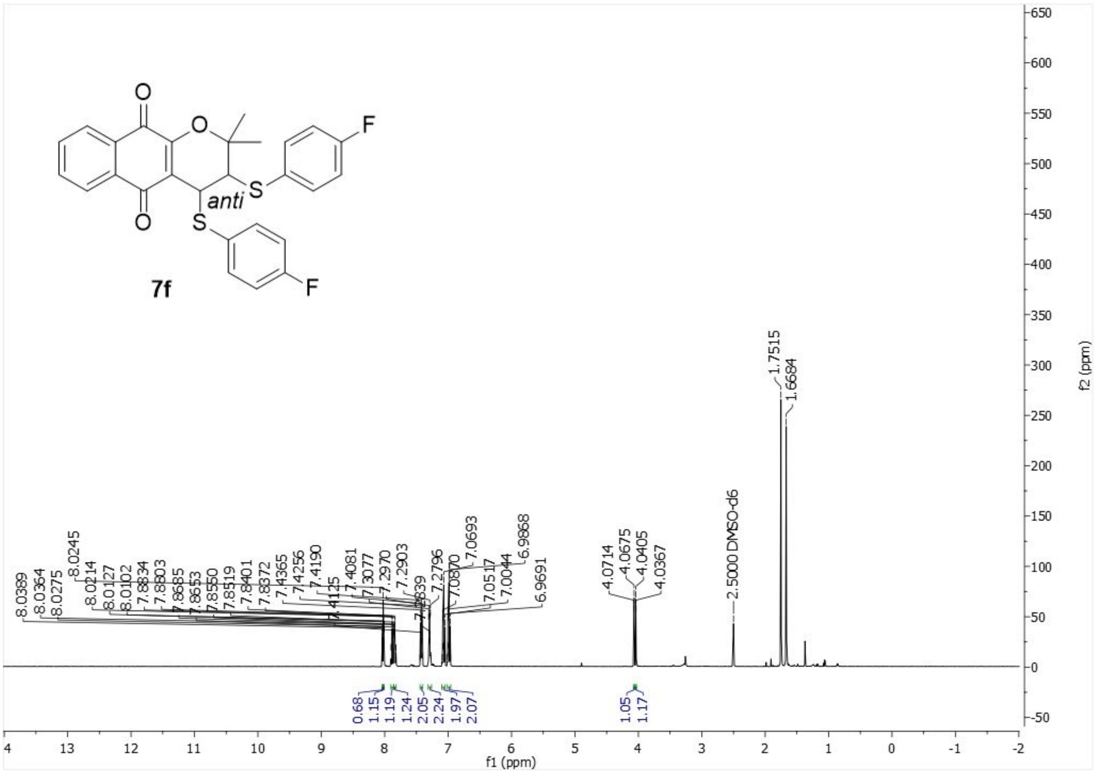

Figure S25.^{1}H NMR (500 MHz, DMSO-d_6) of **7f**

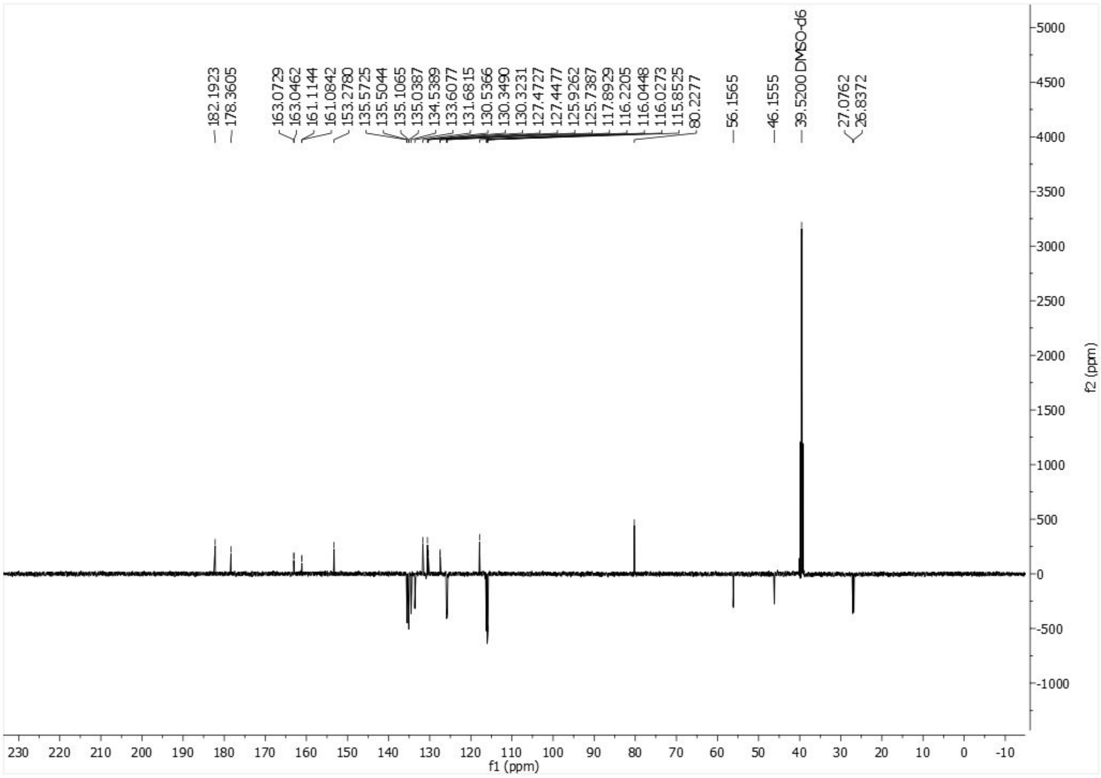

Figure S26.^{13}C/APT NMR (125 MHz, DMSO-d_6) of **7f**

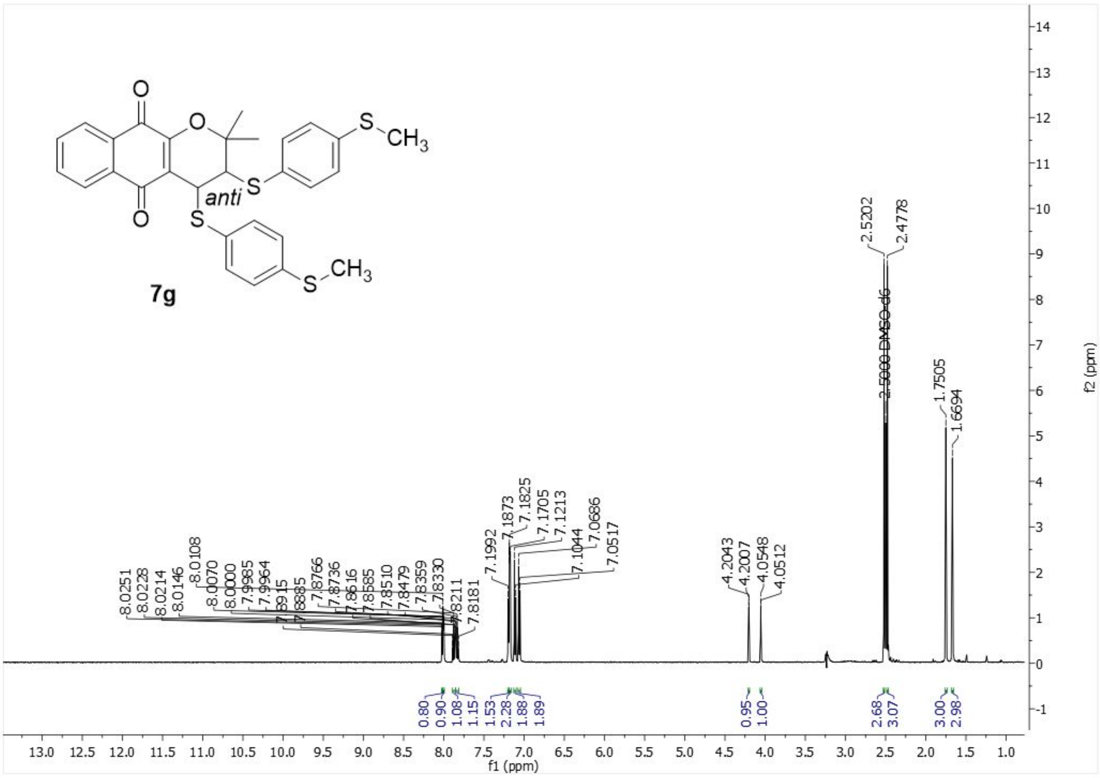

Figure S27.^{1}H NMR (500 MHz, DMSO-d_6) of **7g**

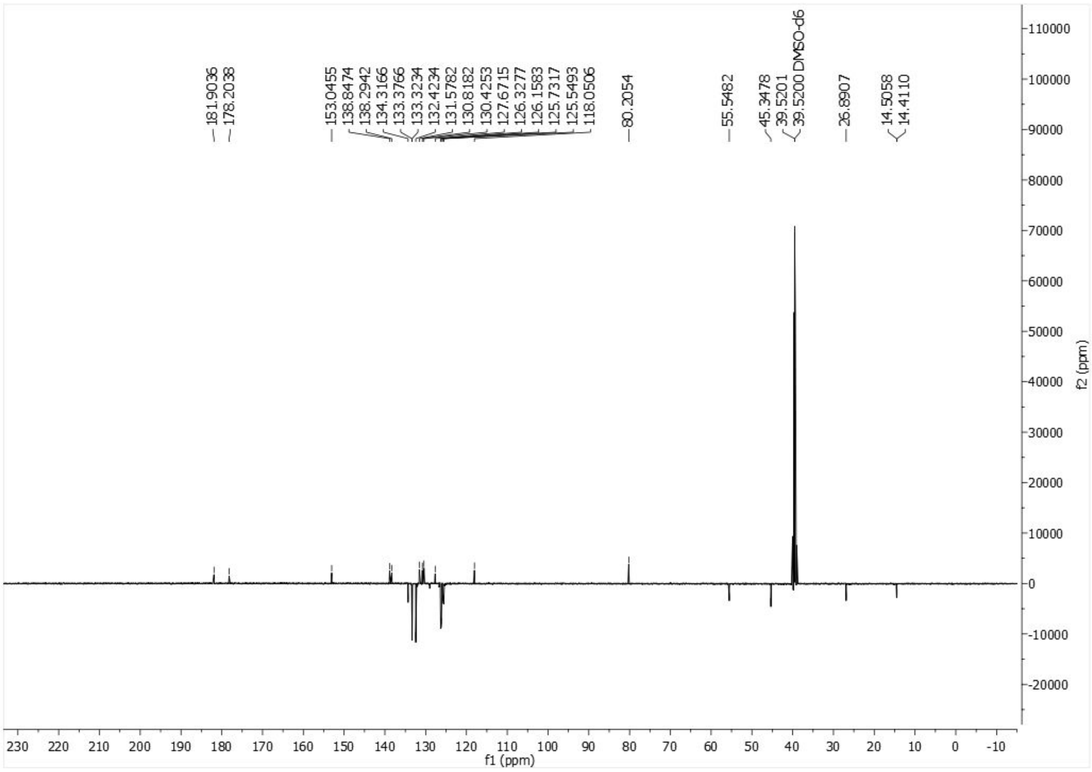

Figure S28.^{13}C/APT NMR (125 MHz, DMSO-d_6) of **7g**

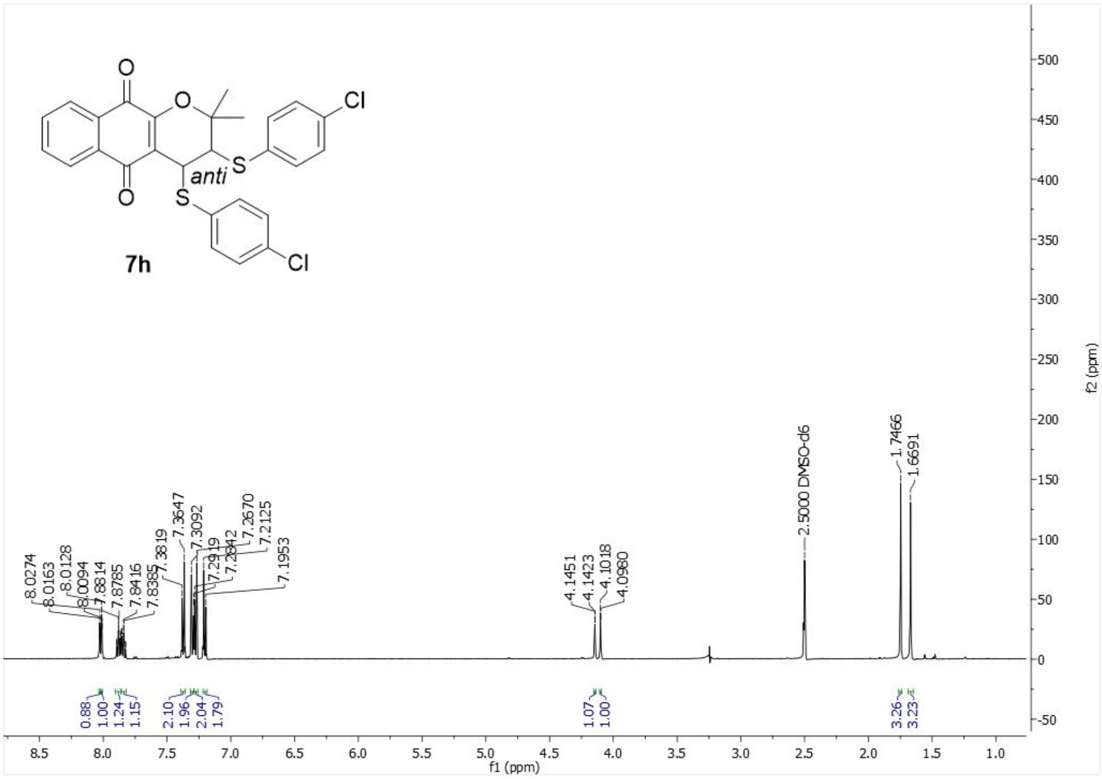

Figure S29. ^{1}H NMR (500 MHz, DMSO-d_6) of **7h**

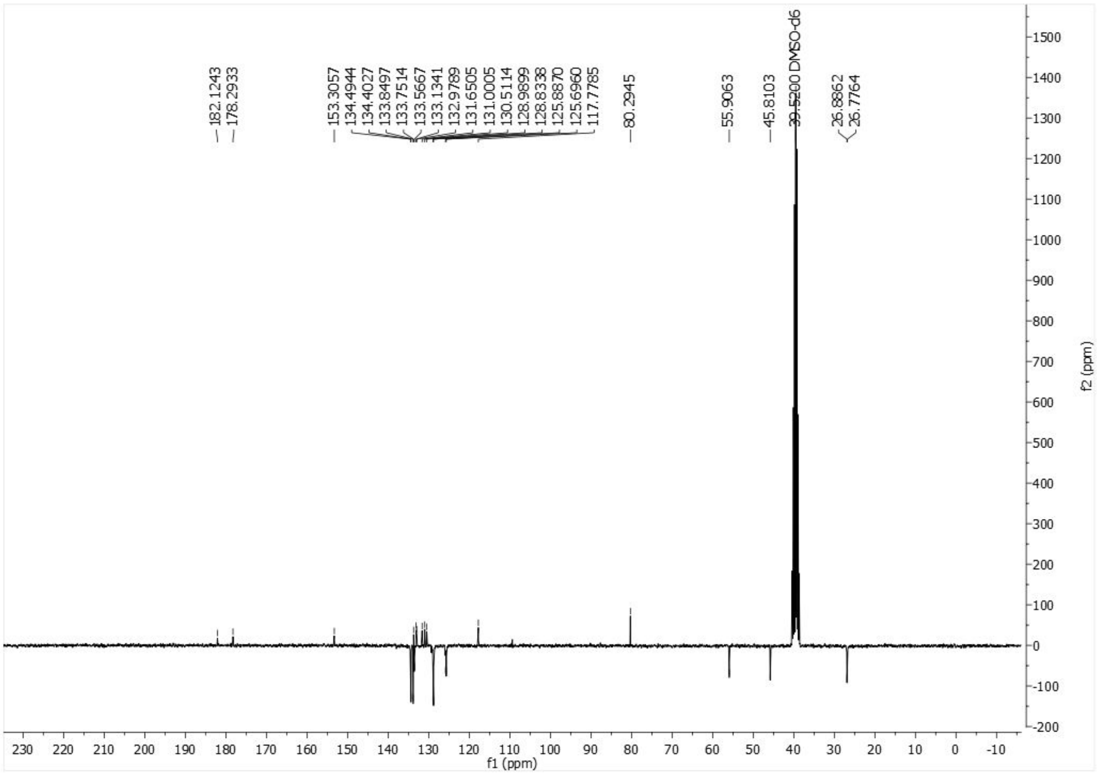

Figure S30.^{13}C/APT NMR (125 MHz, DMSO-d_6) of **7h**

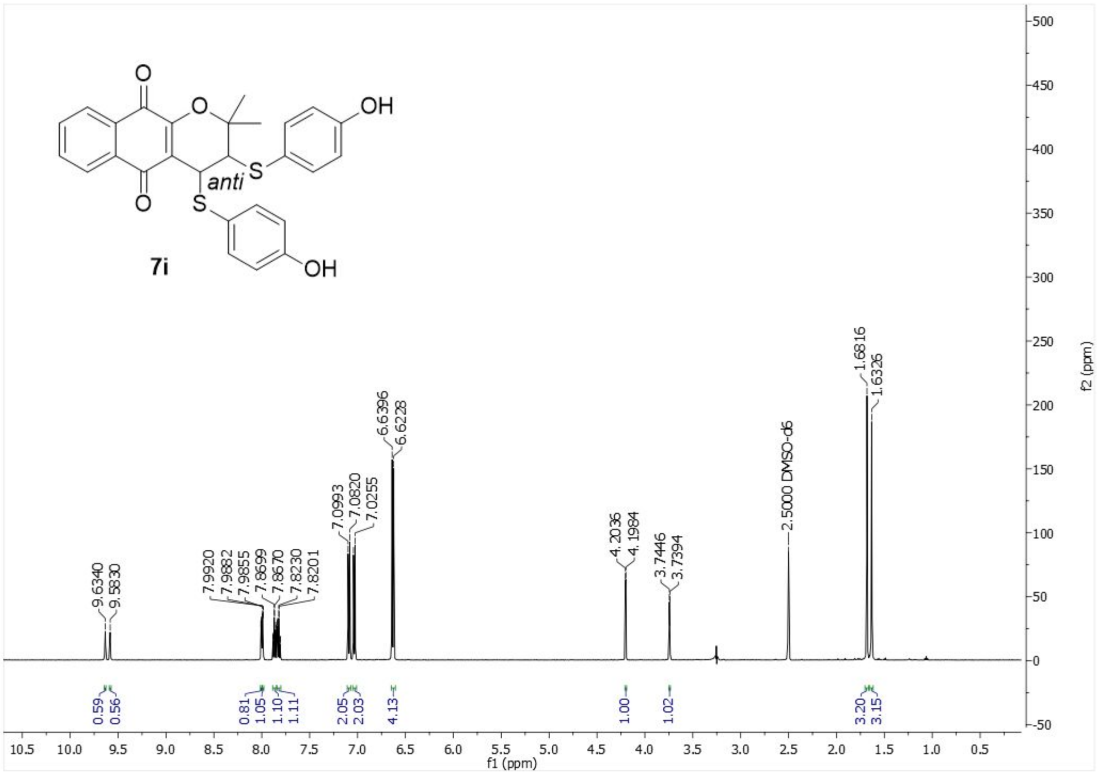

Figure S31.^{1}H NMR (500 MHz, DMSO-d_6) of **7i**

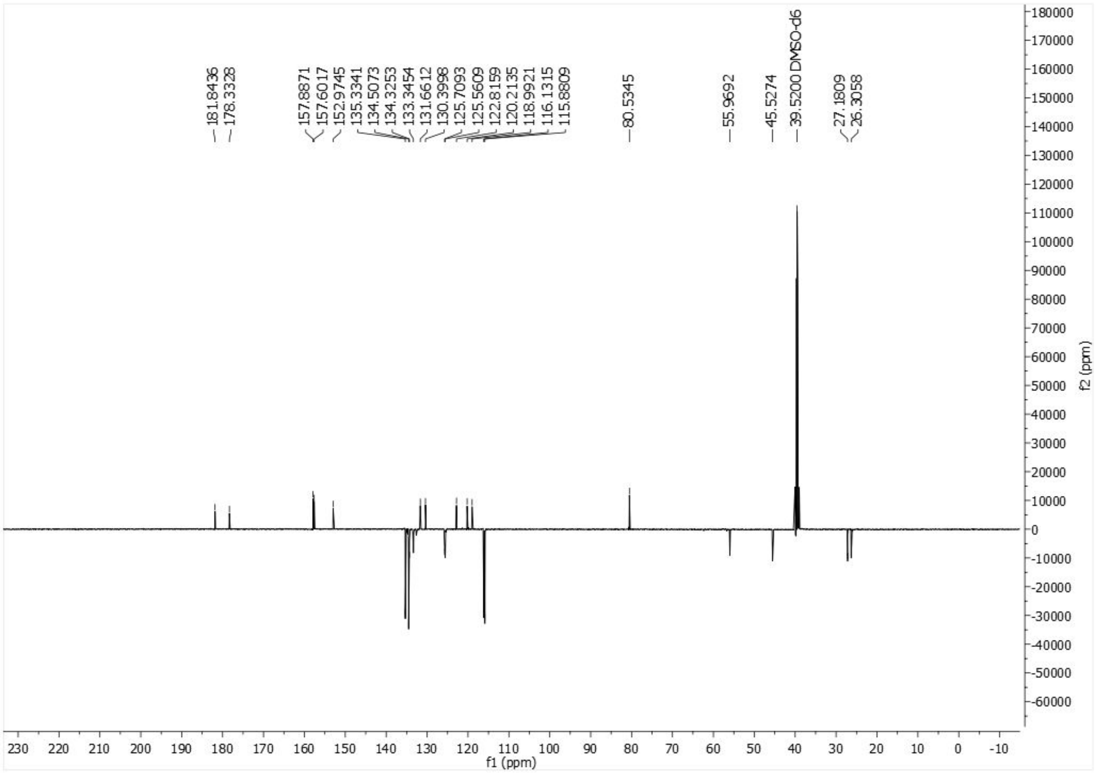

Figure S32.^{13}C/APT NMR (125 MHz, DMSO-d_6) of **7i**

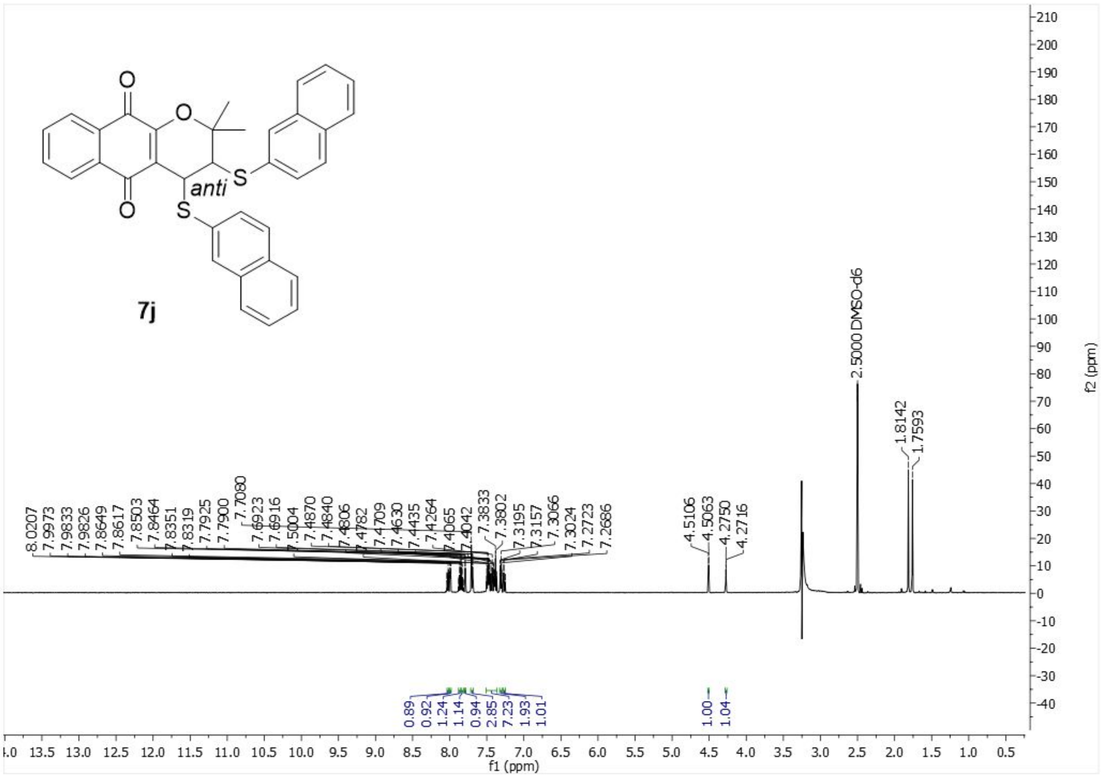

Figure S33.^{1}H NMR (500 MHz, DMSO-d_6) of **7j**

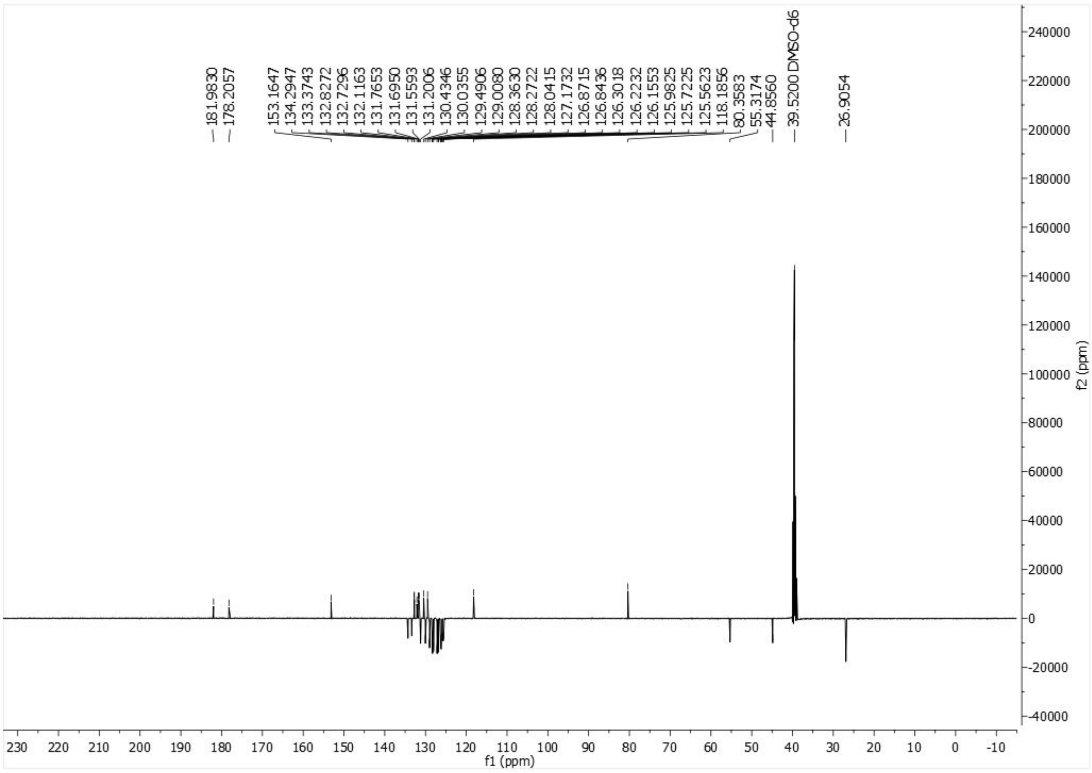

Figure S34.^{13}C/APT NMR (125 MHz, DMSO-d_6) of **7j**
